# A Systematic Review and Bibliometric Analysis of the Scientific Literature on the Early Phase of COVID-19 in Italy

**DOI:** 10.3389/fpubh.2021.666669

**Published:** 2021-06-22

**Authors:** Federica Turatto, Elena Mazzalai, Federica Pagano, Giuseppe Migliara, Paolo Villari, Corrado De Vito

**Affiliations:** Department of Public Health and Infectious Diseases, Sapienza University of Rome, Rome, Italy

**Keywords:** coronavirus, COVID-19, Italy, bibliometric analysis, public health, systematic review

## Abstract

**Background:** Studying the scientific literature about COVID-19 and Italy, one of the first countries to be hit by the pandemic, allows an investigation into how knowledge develops during a public health emergency.

**Methods:** A systematic review of the literature was conducted to identify articles published on the topic between January and April 2020. Articles were classified according to type of study. Co-occurrence of terms, and geographic and temporal trends were analyzed.

**Results:** Of the 238 articles included in the systematic review, the majority (37%) focused on hospital and clinical management of COVID-19, while 23.9% were commentaries. Epidemiological studies constituted 45.5% of the articles published by authors with non-Italian affiliations.

**Conclusion:** The scientific articles on COVID-19 in Italy were varied in type of study, though with limited international impact. The lockdown and the pressure placed on hospitals during the first wave of the pandemic mainly resulted in publications on disease management and commentaries.

## Introduction

Since the first cases of COVID-19 were reported in December 2019 in Wuhan, China (1), the SARS-CoV-2 virus has continued to spread. The World Health Organization (WHO) declared COVID-19 to be a Public Health Emergency of International Concern (PHEIC) by January 30, 2020 (2) and a pandemic by March 11, 2020 (3). In the following months the virus has spread all over the world (4). Italy was one of the countries first affected and, with 1,770,149 confirmed cases and 61,739 COVID-19-related deaths (as of 11 December 2020) (5), it is one of the hardest hit. Italy entered a national lockdown on March 9^th^ (6), which lasted over 2 months, but in March 2021 it is still dealing with COVID-19 and many areas across the peninsula are experiencing new lockdowns (7).

During a pandemic, circulation of information is one of the main weapons allowing the organization of a coordinated response in different countries facing the same emergency (8). This has led many journals to speed up the process of peer review and publication in order to provide large amounts of accessible information to the scientific community and the general public (9, 10). Since much of the scientific literature on the pandemic has been produced in a short time span, it is important to describe and understand the nature of this output and the main elements that characterize it (11). A combination of qualitative analysis and quantitative bibliometric analysis is an effective approach to the analysis of the large amount of scientific literature produced and the identification of the main messages (12). For this purpose, many bibliometric tools such as VOSviewer have been used to investigate the global status and trends of the pandemic (13, 14), to make comparisons among countries (15) or to analyze the scientific output of a single country (16). Italy represents a unique case study: it was the first European country to be hit by the pandemic, and the consequences of the outbreak had a shocking impact on the population. The experience of the Italian hospitals and territories, given their arduous struggle with the pandemic, drew the attention of the entire scientific community. Analysing the scientific literature on COVID-19 and Italy in the first pandemic wave can therefore help us to understand how the scientific output evolves as a new public health threat emerges.

The aim of this systematic review is to describe the key features of the peer-reviewed scientific literature on the COVID-19 outbreak in Italy over the first 4 months of the epidemic (up to April 24, 2020) using both a qualitative and a quantitative approach.

## Methods

A systematic search of the literature was performed using Scopus and PubMed databases on the 24^th^ of April 2020. A comprehensive search strategy was developed to identify articles published since December 2019 which included the terms (“covid” OR “SARS-CoV-2” OR “coronavirus”) AND (“Italy” OR “Italian”) in their title and/or abstract. In order to be included in our study, articles had to address the COVID-19 pandemic in the Italian setting, with no restriction based on language or study design. This systematic review was conducted in accordance with the Preferred Reporting Items for Systematic Reviews and Meta-Analyzes (PRISMA) 2009 Statement (17), although we do not present the characteristics for each article included, as it is beyond the scope of the study.

Retrieved articles were then evaluated independently by three researchers to ensure only articles related to the current SARS-CoV-2 pandemic in Italy were included in the analysis. For each included item, publication date, title, journal, first author's gender and first author's nationality of affiliation (Italian or non-Italian) were extracted. For Italian publications, region of first author's affiliation was determined; for non-Italian publications, country of first author's affiliation was determined. When the first author was affiliated to a research center managed by different regions, such as IRCCS (Istituto di Ricovero e Cura a Carattere Scientifico), we assigned the region according to where the institute is based. Gender was not assigned when the author was an institution and for authors where gender could not be inferred. Region/country of affiliation was not assigned when the affiliation was a national institution, a journal or a scientific society. The impact factor for each journal was obtained from the Journal of Citations Report 2019 (18).

Articles were classified according to study type based on the classification of studies in medical research developed by Röhrig et al. (19), which was expanded and adapted for the purposes of this study. The type of study was defined according to the contents, rather than its form of publication (e.g., commentaries including case reports were classified as case reports rather than commentaries). The following categories were added by the researchers to the original classification by Röhrig et al. (19): *Modeling and prediction* included studies in which mathematical models were developed to make predictions about the pandemic; the *Management* category included *Hospital management case report* (accounts of hospital management strategies undertaken to combat the pandemic, for example, reorganization of wards, reallocation of HCWs), *Clinical management case report* (accounts of algorithms used to manage COVID-19 patients) and *Experts' recommendation* (recommendations on hospital and/or clinical management issued by scientific societies or groups of experts); the category defined as *Other* included *Ethics and Legal Medicine* **(** considerations on ethical or legal aspects relating to decision-making during the pandemic), *Commentary and Viewpoint* (generic considerations without original information).

VOSviewer (version 1.6.15) was used to perform co-occurrence analyzes on terms from titles and abstracts in order to visualize the main topics of the publications. Co-occurrence analysis reveals how often two words appear together in the same text as well as the connections between terms. In the resulting visual network, each sphere represents a term, and the size of the sphere is proportional to the occurrence of the term. The links between the spheres represent the association between words: the thicker the line, the stronger the association (co-occurrence). The program identifies clusters of words that are very often cited together and likely refer to the same topic. Two co-occurrences analyzes were performed: one including words from both title and abstract and one considering words from abstract only. The occurrence threshold for our study was set at five, with an automatic selection of 60% of co-occurring words based on relevance. Time trends, geographical analyzes and journal analyzes were carried out in Microsoft Excel.

## Results

**Figure 1:**
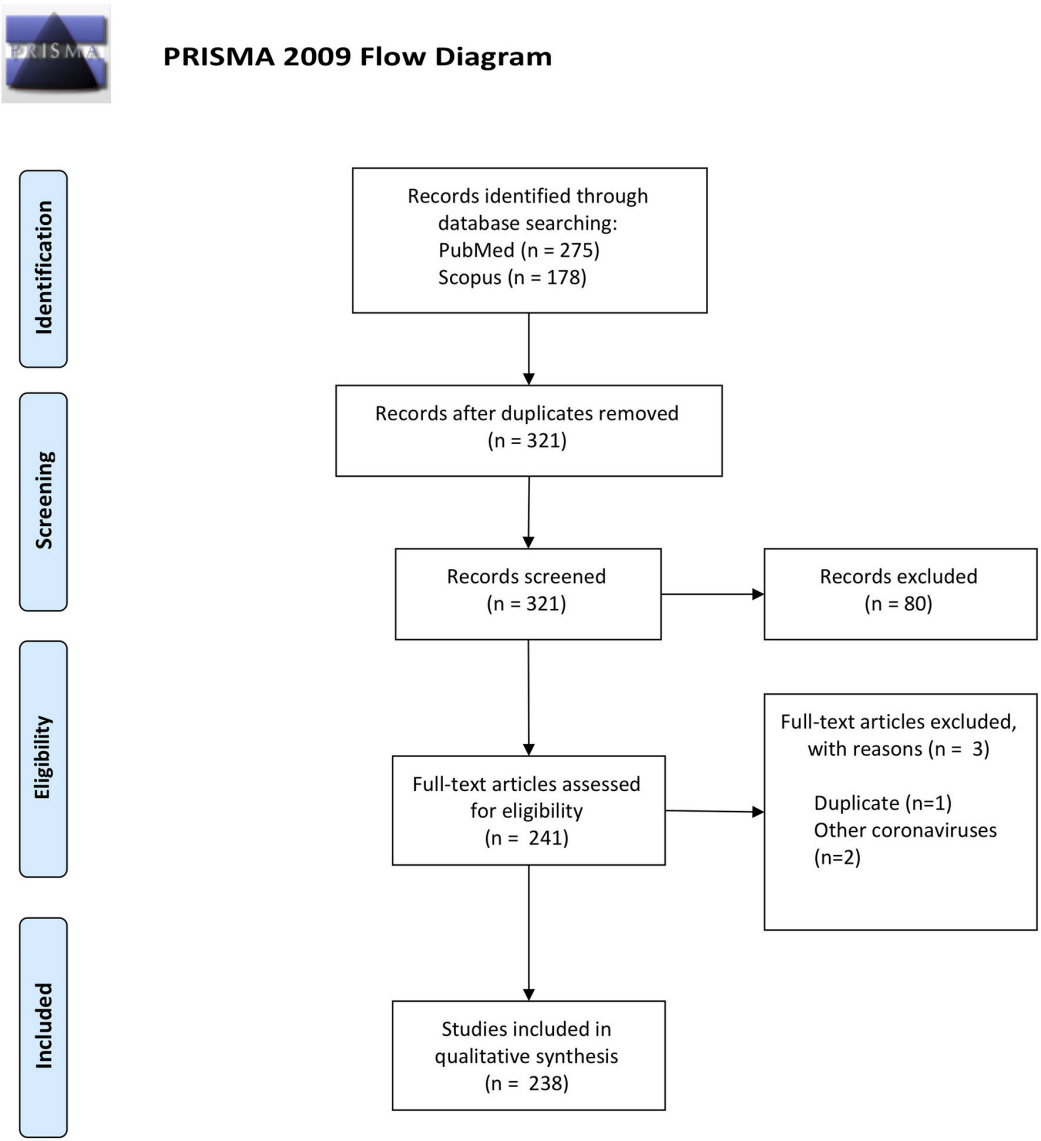
PRISMA diagram of the study selection process.

Of the 321 studies retrieved from the search (Figure 1), 238 articles were included in the analysis: 205 where the first author's affiliation was with an Italian institution and 33 where the first author had a non-Italian affiliation. [From this point, studies with a first-author Italian affiliation will be called “Italian” studies, with the others being called “non-Italian” studies].

### Content Analysis

**Figure 2:**
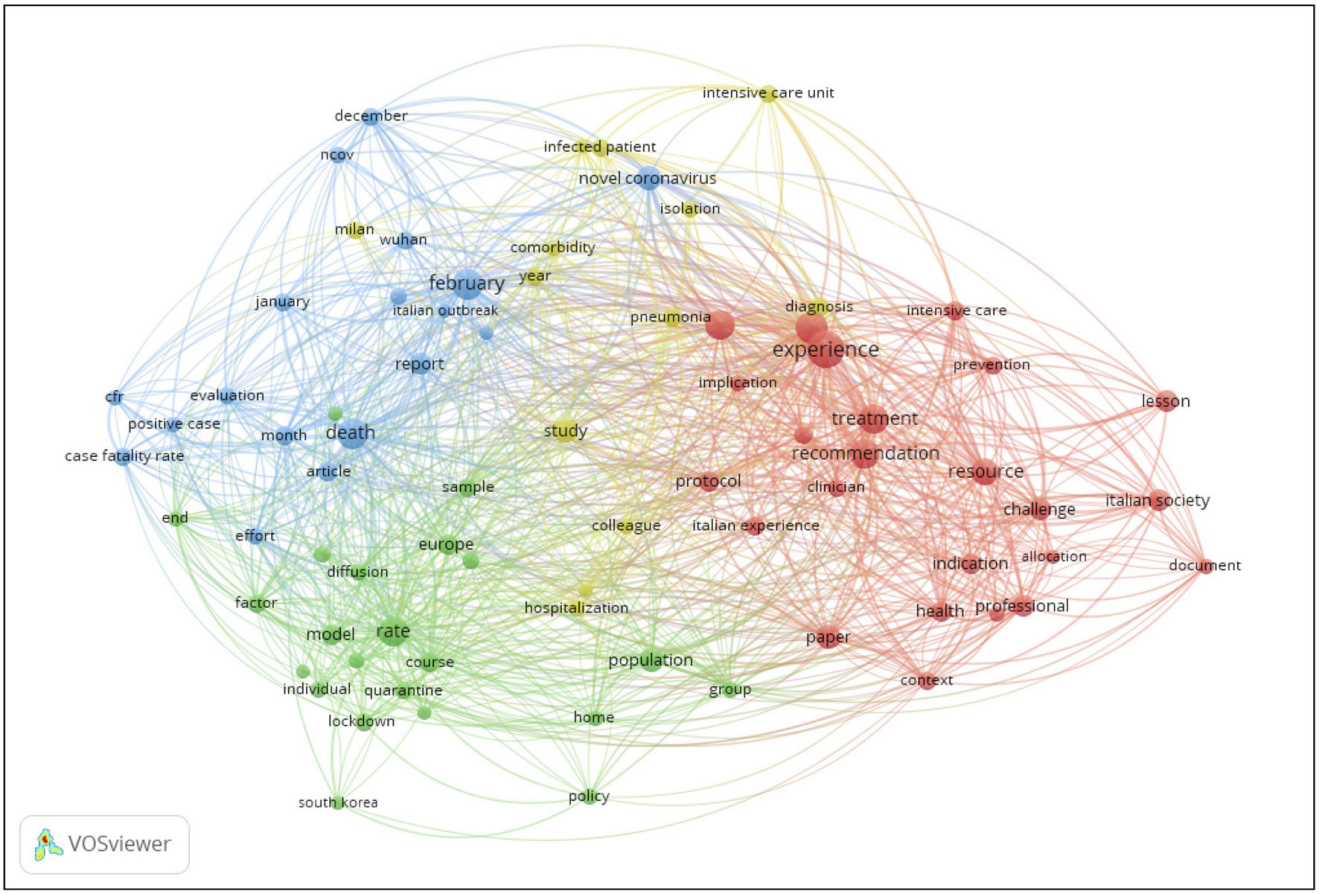
Co-occurrence analysis using VOSviewer of terms in titles and abstracts of Italian studies.

Abstracts were not available for 108 out of 205 Italian and 22 out of 33 non-Italian articles. For these studies, only words included in the title were analyzed with the VOSviewer software for the co-occurrence analysis performed on title and abstract. Based on this analysis, four clusters emerged, highlighted in different colors (Figure 2): red cluster, labeled as “hospital and clinical management,” containing 24 items; blue cluster, labeled as “descriptive epidemiology,” containing 22 items; green cluster, labeled as “policies and public health,” containing 18 items; yellow cluster, labeled as “generic,” with transverse items not specific to other clusters, containing 13 items. The most cited words were: “experience” (36 occurrences), “management” (27 occurrences), “February” (25 occurrences) and “death” (25 occurrences).

A further analysis based on abstracts only was performed. The resulting network is shown in Supplementary Figure 1 and includes four clusters: red cluster, labeled as “hospital management”, containing 16 items; blue cluster, labeled as “clinical management,” containing 12 items; green cluster, labeled as “epidemiology,” containing 12 items; yellow cluster, labeled “generic,” with transverse items not specific to other clusters, containing 12 items.

### Classification of the Retrieved Articles

Articles were classified according to study type, using Röhrig's classification (19) as baseline (Table 1). Half of the Italian publications were classified as either *Hospital management case report* (55) or *Commentary and Viewpoint* (48) (103 out of 205, 50.2%). Non-Italian publications were more equally distributed: 21 out of 33 (63.6%) were either *Commentary and Viewpoint* (nine), *Monitoring and Surveillance* (eight) or *Narrative review* (four). All the *Basic* research studies identified (five Italian and two non-Italian) were *Genetic engineering and Gene sequencing* articles. Italian *Clinical* research studies were mostly observational (26), and only one was experimental, while no *Clinical* studies were found among non-Italian articles. Among the observational epidemiological studies, Italian articles mainly reported results of *Monitoring and Surveillance* and *Modeling and Prediction* studies (eight and six, respectively), while the non-Italian were mostly *Monitoring and Surveillance* (four) and *Ecological study* (four) articles. Articles assigned to the *Management* category were mainly *Hospital management case report (*55), *Experts' recommendation* (24) and *Clinical management case report* (nine). No experimental *Epidemiological* studies were found. Among *Secondary* research studies, 10 Italian and four non-Italian *Narrative review* articles and *one* Italian and one non-Italian *Systematic review* articles were found.

**Table 1:** Classification of the 238 retrieved articles by type of study.

				**Italian**		**Non-Italian**	
				***n***	**%**	***n***	**%**
Primary (157)	Basic (7)	Theoretical (method development)	Analytical measurement procedure	0	0	0	0
			Imaging procedure	0	0	0	0
			Biometric procedure	0	0	0	0
			Test development assessment procedure	0	0	0	0
		Applied	Animal study	0	0	0	0
			Cell study	0	0	0	0
			Genetic engineering and Gene sequencing	5	2.4	2	6.2
			Biochemistry	0	0	0	0
			Material development	0	0	0	0
			Genetic studies	0	0	0	0
	Clinical (27)	Experimental	Clinical study	1	0.5	0	0
		Observational	Therapy study	3	1.5	0	0
			Prognostic study	0	0	0	0
			Diagnostic study	3	1.5	0	0
			Observational study with drugs	0	0	0	0
			Secondary data analysis	0	0	0	0
			Case series	15	7.3	0	0
			Single case report	5	2.4	0	0
	Epidemiological (35)	Experimental	Intervention study	0	0	0	0
		Observational	Cohort study	0	0	0	0
			Case control study	0	0	0	0
			Cross-sectional study	2	1	0	0
			Ecological study	2	1	4	12.1
			Monitoring and Surveillance	8	3.9	8	24.2
			Modeling and Prediction	6	2.9	2	6.1
			Description with registry data	2	1	1	3
	Management (88)	Hospital management case report		55	26.8	0	0
		Clinical management case report		8	3.9	0	0
		Experts' recommendation		24	11.7	1	3
Secondary (16)	Meta-analysis			0	0	0	0
	Review (16)	Narrative		10	4.9	4	12.1
		Systematic		1	0.5	1	3
Other (65)	Ethics and Legal Medicine			7	3.4	1	3
	Commentary and Viewpoint			48	23.4	9	27.3
Total				205	100.0	33	100.0

### Geographical Distribution

We compared the geographical distribution of the Italian publications with the distribution of COVID-19 density of cases at the end of the study period (Table 2). The region was not attributable in 13 articles (6.4%).

**Table 2:** Geographical distribution of cases of COVID-19 and articles published up until 24^th^ April 2020. Regions are ordered by decreasing density of cases.

	**Cumulative cases of COVID-19^**a**^**	**Population^**b**^**	**Density of cases (cases per 10.000 inhabitants)**	**N. of articles published**
Valle d'Aosta	1,100	125,034	87,98	0
Lombardy	71,256	10,027,602	71,06	73
Trentino-Alto Adige	6,232	1,078,069	57,81	4
Piedmont	23,822	4,311,217	55,26	10
Emilia-Romagna	23,970	4,464,119	53,69	18
Liguria	7,173	1,524,826	47,04	2
Marche	6,028	1,512,672	39,85	5
Veneto	17,229	4,879,133	35,31	7
Tuscany	8,877	3,692,555	24,04	11
Friuli Venezia Giulia	2,882	1,206,216	23,89	6
Abruzzo	2,803	1,293,941	21,66	2
Umbria	1,363	870,165	15,66	0
Lazio	6,132	5,755,700	10,65	33
Apulia	3,881	3,953,305	9,82	4
Molise	287	30,0516	9,55	0
Sardinia	1,257	1,611,621	7,80	4
Campania	4,282	5,712,143	7,50	7
Basilicata	360	553,254	6,51	0
Sicily	2,981	4,875,290	6,11	3
Calabria	1,079	1,894,110	5,70	3

a *Source of data: Italian Civil Protection Department (20)*.

b *Population at January 1, 2020. Source of data: National Institute of Statistics (ISTAT) (21)*.

By April 24^th^ Italy had accumulated 192,994 cases (20). Density of cases per region showed a clear north-south gradient (Supplementary Figure 2). Of the 205 Italian articles, 73 (35.6%) were published in Lombardy, which was the second region for density of cases, while 33 (16.1%) were published in Lazio, which was among the regions with the lowest density of cases (Table 2). The analysis of the characteristics of the type of study showed that *Management* studies were published mainly in Lombardy (42), Emilia-Romagna (nine) and Lazio (seven). *Clinical* studies followed a similar pattern, with 12 publications from Lombardy and eight from Lazio, while *Commentary and Viewpoint* articles were more equally distributed among regions, as were *Epidemiological* studies (Supplementary Figure 3).

The 33 non-Italian articles were published in fifteen countries. Nine were published by authors based in the United Kingdom, four in the United States and Sweden, three in China, two in Brazil and two in Iran.

### Trends in Type of Publication

**Figure 3:**
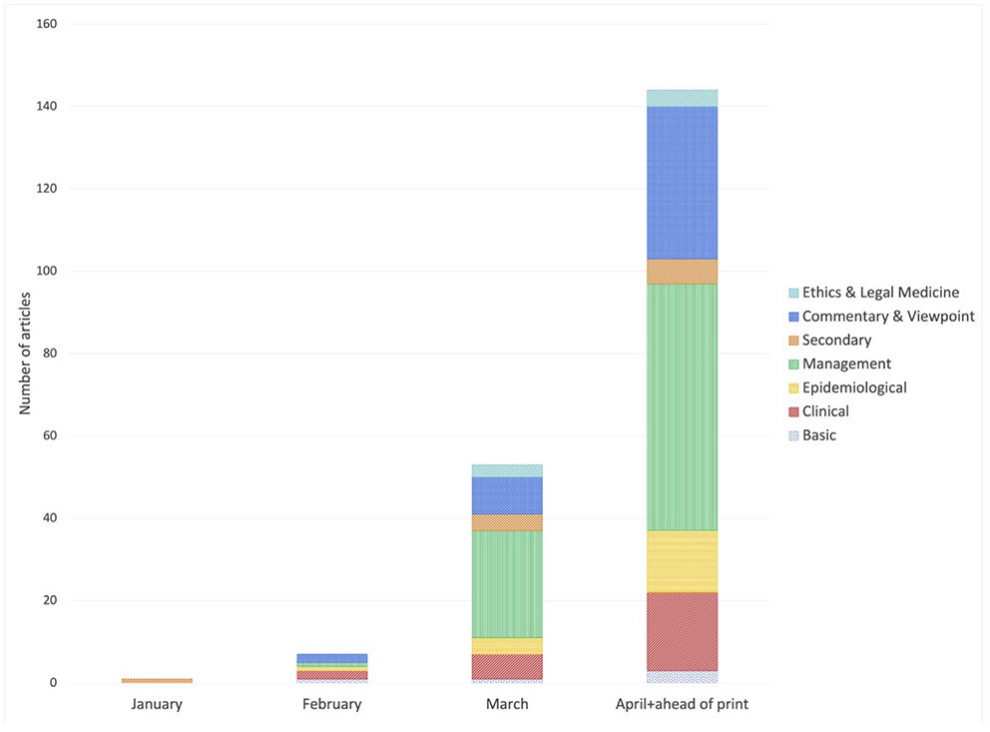
Italian articles published each month, by type of study.

There was a marked increase in publications over time as the pandemic progressed, beginning with a single article published in January to 144 articles published in April. The first type of publication to appear was a *Narrative review* in January, after which various types of articles were published in February, although in small numbers: one *Basic* study, two *Clinical* studies, one *Epidemiological* study, one *Secondary* study, one *Management* study and two *Commentary and Viewpoint* articles. From March onward, the number of publications in each category, especially *Management* studies, increased. *Ethics and Legal Medicine* articles started to appear in March (three) and April (four) (Figure 3). *Management* articles increased in absolute numbers and in percentage, making up half of the publications in March, then slightly decreased in April by percentage, but not in absolute numbers. *Commentary and Viewpoint* articles emerged relatively early and remained more or less stable though time (28.6% in February, 17.0% in March and 25.7% in April), with an increase in absolute number month by month.

**Figure 4:**
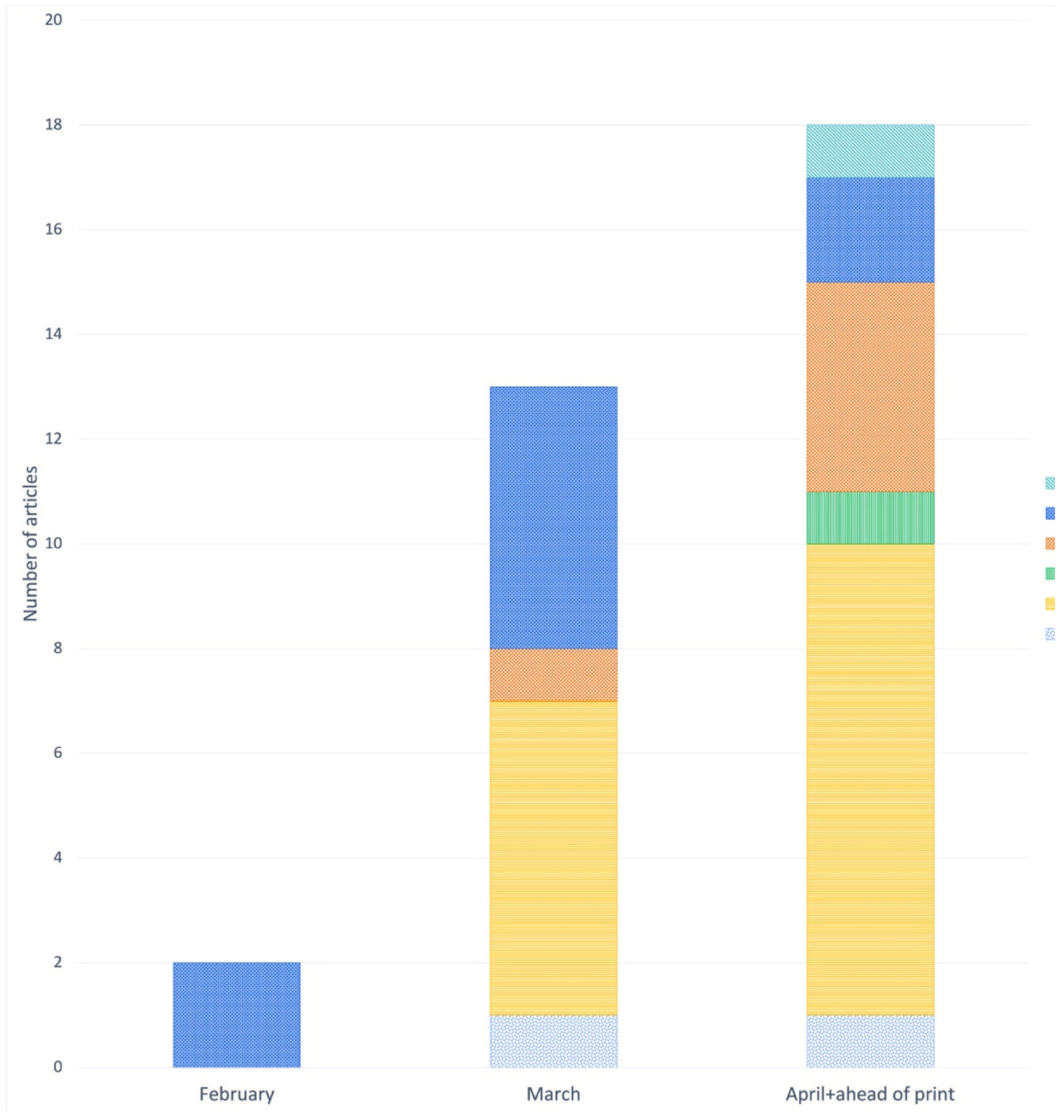
Non-Italian articles published each month, by type of study.

Non-Italian publications started to appear in February with two articles categorized as *Commentary and Viewpoint*. In March and April, there was an increase in the number and variety of articles. The most represented category, appearing in March and increasing in April, was *Epidemiological* studies (six and nine articles, respectively). We found only one *Management* report, published in April (Figure 4). The proportion of *Commentary and Viewpoint* articles decreased with time, with a simultaneous increase in the other types of publication.

### Journals

**Figure 5:**
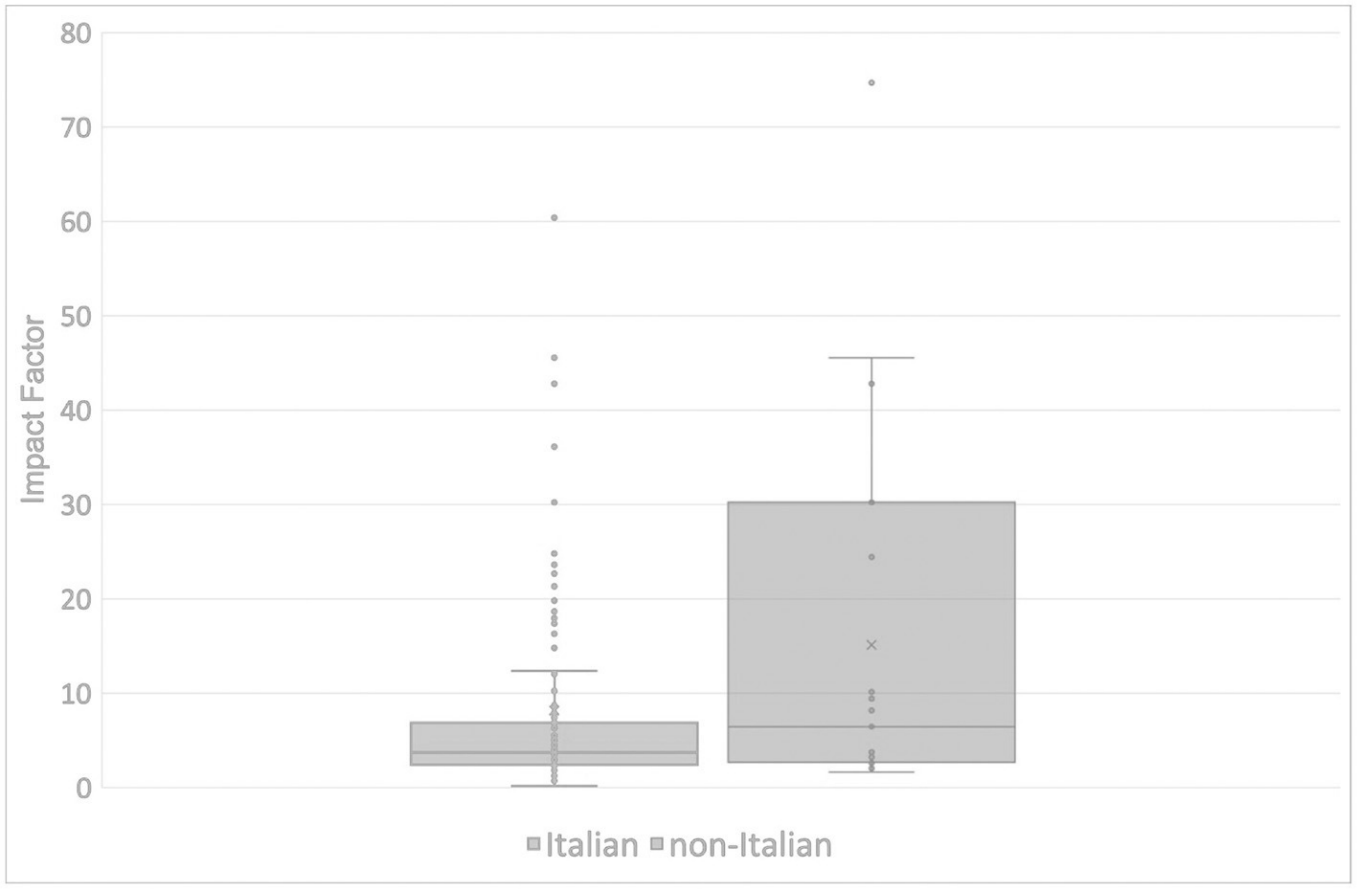
Distribution of Impact Factor of the journals that published Italian (left) and non-Italian (right) articles.

The Italian articles were published in 153 different journals. Among these, 30 journals published more than one article each and five journals more than three. In particular, seven articles appeared in the *Journal of Medical Virology*, five in *The Lancet* and *Giornale Italiano di Nefrologia*, and four in *Eurosurveillance* and *Recenti Progressi in Medicina*. Two of these five journals do not have an impact factor (IF) according to the Journal of Citations Report 2019. Four of the top ten journals ranked by IF published at least two articles. *The Lancet*, the journal with the highest IF (60.39), published five articles, followed by *JAMA* (45.54) with three publications. Globally, 18 articles were published in the top ten journals by IF. The median IF score for Italian publications was 3.75, with a mean of 8.16 (Figure 5).

Non-Italian articles were published in 25 different journals. Three journals published more than one article: *The BMJ—British Medical Journal* published five articles, *Eurosurveillance* and *Journal of Medical Virology* three articles each. Ranked by IF, the first three journals (*The New England Journal of Medicine* IF 74.699, *JAMA* IF 45.54 and *Nature* IF 42.78) had one publication each. The median IF and the mean IF of the non-Italian journals were 6.46 and 15.11, respectively (Figure 5).

## Discussion

The number of articles published globally relating to the pandemic has grown exponentially since the first cases were confirmed in China. An analysis carried out on PubMed on the 25^th^ of April by Kambhampati and colleagues detected 6,831 articles on the pandemic and showed an exponential growth of publications relating to COVID-19 since the beginning of the year (22). Our review focused on a specific portion of the global literature on the pandemic, that is, those publications pertaining to Italy. Accordingly, our search yielded mainly articles published by authors with an Italian affiliation, and a smaller number of articles published by non-Italian authors that included Italy in wider analyzes.

Due to its early involvement in the current pandemic, Italy has scaled up its contribution to research in the field of coronaviruses (23). During the SARS and MERS outbreaks, Italy did not appear in the top 10 contributing countries (24) and, according to the literature, the Italian share of the global scientific production on COVID-19 was itself limited up to the end of February 2020 (25). However, by the end of March, Italy's contribution amounted to almost 7% of global output (26), and increased further to 7.6% by the end of April (27) and to almost 9% by the beginning of May 2020 (28).

The regional distribution of the scientific output from Italy is comparable to the distribution of COVID-19 density of cases reported in the different regions, with some exceptions: the Lazio region released a relatively high number of publications given its share of cases, but this can be explained by the presence of national research institutes in this region. On the other hand, other regions with a high density of cases, like Veneto and Piedmont, published relatively few articles. As might be expected, the hardest hit region at the time (Lombardy) published a proportionally large number of articles relating to the management of the outbreak and to clinical aspects, thus illustrating the differing impact of the pandemic across the country.

The analyzes carried out with VOSviewer showed that the main themes were the epidemiology of the disease and the management of the outbreak in hospital settings. The focus of many studies on management aspects of the pandemic was confirmed in our analysis when articles were classified by study type, which revealed that most articles with an Italian affiliation consisted of hospital management case reports and commentaries. Our resulting map (Figure 2) differs from similar analyzes of the global literature using keywords carried out on VOSviewer, which showed a wider prevalence of clinical terms (29–31). A focus on clinical aspects related to COVID-19 was also found in an analysis of Iranian publications (16). The content analysis carried out on abstracts only did not identify a policy field. This is probably due to the fact that articles dealing with policy aspects were mainly commentaries and viewpoints, which were not always provided with an abstract.

All types of publication increased with time, with a notable increase in the share of articles relating to the management of the pandemic, which mainly comprised hospital management case reports and experts' recommendations, in March and April. This might reflect the need to share experience accumulated in the field through publication. The increase in the number of publications that aim to provide expert consensus on COVID-19 management has raised concerns with some authors, due to the lack of evidence underlying such recommendations (32). Non-Italian articles showed a different publication pattern: most were epidemiological studies, followed by commentaries and narrative reviews, while there were, unsurprisingly, relatively few management reports due to our search strategy.

With respect to original research, Chahrour et al. (33) have pointed out that until mid-March 2020 the Italian contribution was small compared to the number of cases of COVID-19 in the country. This was confirmed by Nowakowska et al. (26), who quantified the Italian contribution as 3.2% of the global output of original research by the end of March 2020; Chinese authors were the most prolific, with a 57.7% share of published articles. Our analysis of Italian output shows that there was an increase in basic, epidemiological and clinical research publications in March and especially in April 2020. Part of what we observed could be due to the fact that the first cases in Italy were identified in late February 2020, 2 months after the outbreak in China. It should also be noted that in our analysis we classified the articles according to their content rather than the format of publication. Since many articles were published as letters to the editor or commentaries in order to speed up the publication process, even when they contained original information (26), classifications based on format of publication could lead to an underestimation of the contribution to original research. As Zhai et al. (23) have shown, the number of articles published as letters was also relatively high during the year of the MERS and SARS outbreaks, and then decreased in the following years.

By analyzing the journals and impact factors, we found that, overall, non-Italian articles were published in journals with higher impact factor than Italian articles. This could be due to the need for Italian authors to share knowledge with a small circle of colleagues who faced the same challenges within the country. This hypothesis is supported by the prevalence of management publications. In contrast, non-Italian articles usually included Italy in broader epidemiological analyzes and were addressed to a wider public.

It is interesting to note that women constituted only a small proportion of the first authors of the articles retrieved in our analysis. The proportion was remarkably low for Italian articles (22%) compared to non-Italian articles (48.5%). Further analyzes could clarify whether there has been a decline in the number of female first authors in Italy with the pandemic, as has been shown by Andersen and colleagues for global medical output (34).

We acknowledge some limitations to our analysis. First, we searched only the PubMed and Scopus databases, thereby potentially underestimating the number of publications. Second, since less than half of the articles included in the analysis had an abstract, VOSviewer mainly considered terms included in the titles, which could have provided a less sensitive analysis of the content of the studies. Finally, our study was limited to items published up to 24^th^ April 2020, and therefore provides only an initial overview of the contribution of Italian publications to the growing body of scientific output on COVID-19. Indeed, a bibliometric analysis of global scientific output of COVID-19 carried out in June 2020 already showed that the Italian contribution had grown to 12.2% (31).

To our knowledge, however, this is the first study to comprehensively evaluate scientific publications on COVID-19 in Italy, the first country in Europe to be hit by the pandemic. We believe this analysis provides an important starting point for understanding the mechanisms of dissemination of knowledge in critical times such as the current COVID-19 pandemic.

## Data Availability Statement

The original contributions presented in the study are included in the article/Supplementary Material, further inquiries can be directed to the corresponding author/s.

## Author Contributions

FT and EM equally contributed to the literature searches, data extraction, data analysis, and drafting the manuscript. FP contributed to the data extraction. GM contributed to the bibliometric analysis. PV and CD conceived the study. CD contributed to the planning of the work, reviewed, and edited manuscript drafts. All authors contributed to the design of the study, revised, and approved the final version of the manuscript.

## Conflict of Interest

The authors declare that the research was conducted in the absence of any commercial or financial relationships that could be construed as a potential conflict of interest.
