# Supplementary material for: A Systematic Review and Bibliometric Analysis of the Scientific Literature on the Early Phase of COVID-19 in Italy
Source: Front Public Health. 2021 Jun 22;9:666669. doi: 10.3389/fpubh.2021.666669 (PMC8258167; doi:10.3389/fpubh.2021.666669)
Supplement: Supplementary file 1 [file Data_Sheet_1.pdf]

Supplementary Figure 1. Co-occurrence analysis using VOSviewer of terms in abstracts of Italian studies.

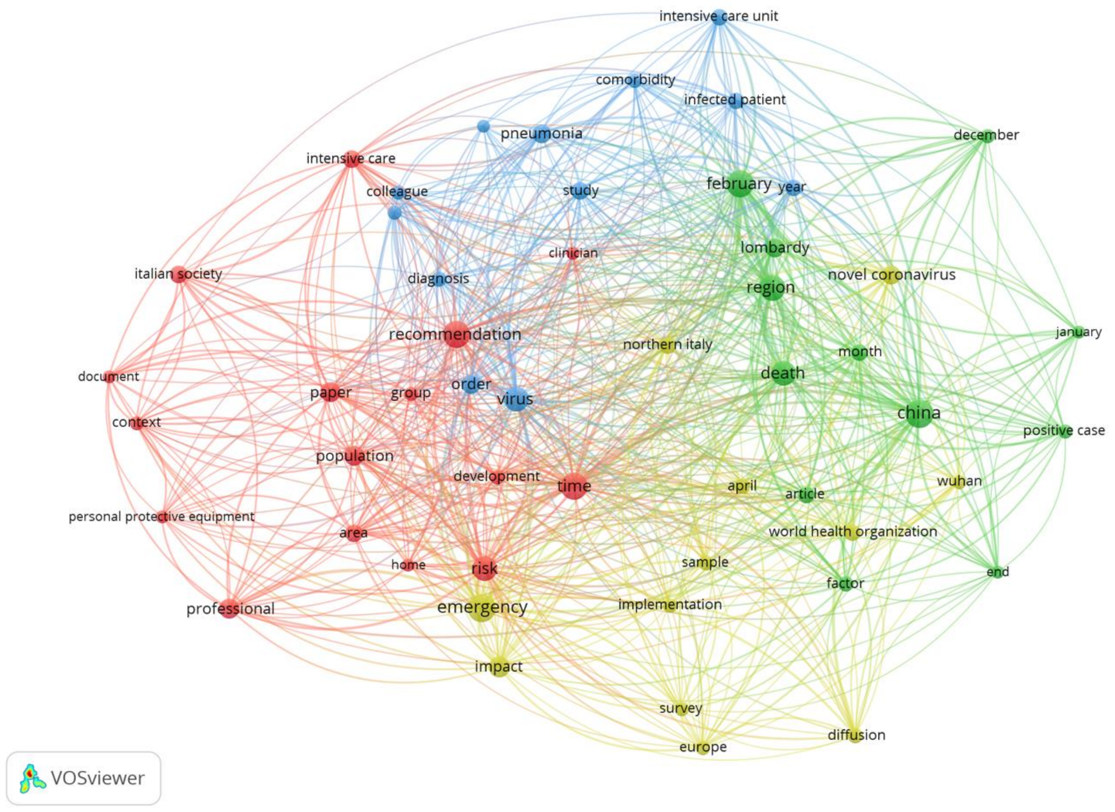

Supplementary Figure 2. Geographical distribution of the Italian publications (A) and density of COVID-19 cases (B) at the end of the study period.

(A)

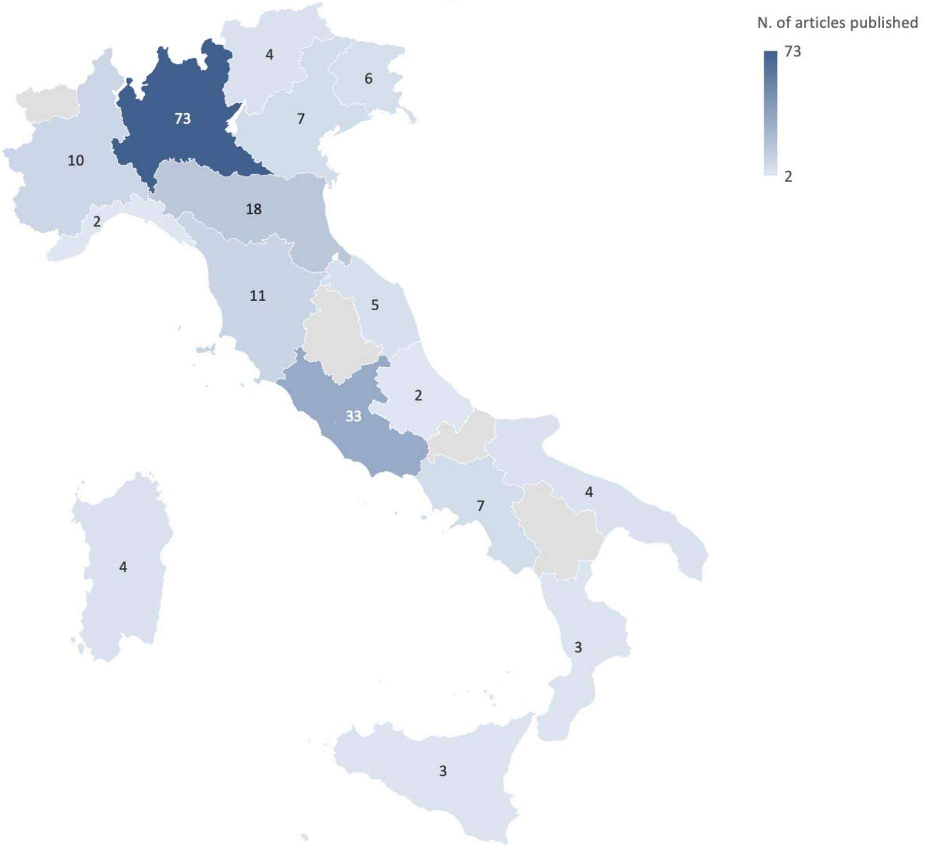

(B)

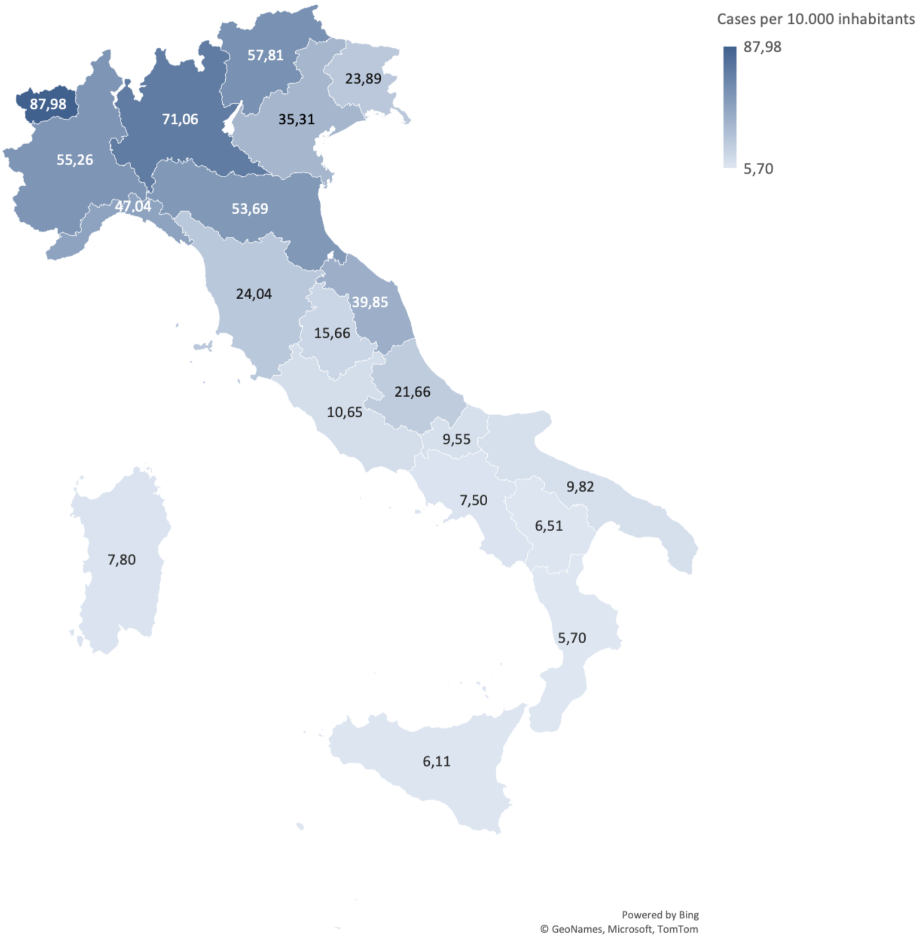

Supplementary Figure 3. Italian articles published in each region, by type of study.

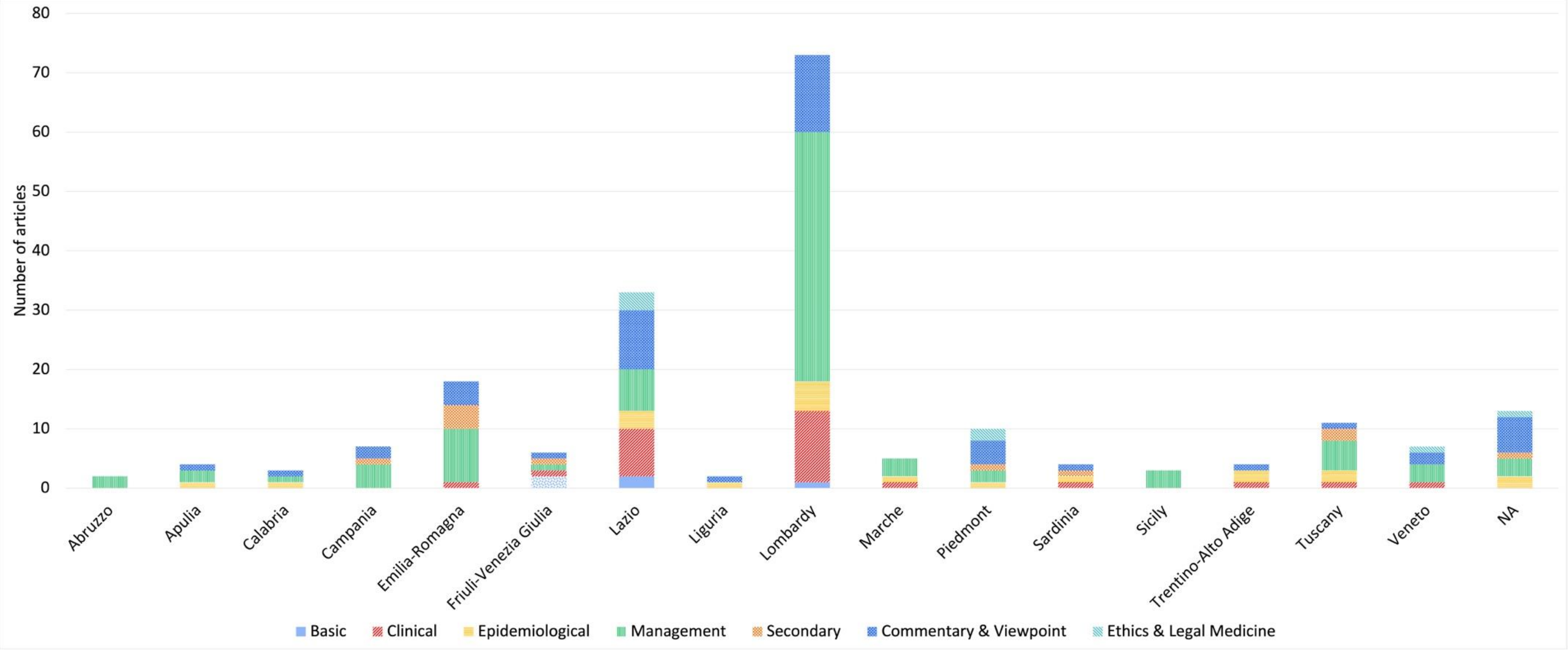

Italian articles included in the study

1. Abenavoli L, Cinaglia P, Luzza F, Gentile I, Boccuto L. Epidemiology of Coronavirus Disease Outbreak: The Italian Trends. Reviews on recent clinical trials [Internet]. 2020; Available from: https://unigastro.it/Allegati/Epidemiology%20of%20Coronavirus%20Disease%20Outbreak%20The%20Italian%20Trends.pdf

2. Agostini A, Floridi C, Borgheresi A, Badaloni M, Esposto Pirani P, Terilli F, et al. Proposal of a low-dose, long-pitch, dual-source chest CT protocol on third-generation dual-source CT using a tin filter for spectral shaping at 100 kVp for CoronaVirus Disease 2019 (COVID-19) patients: a feasibility study. Radiologia Medica. 2020;125(4):365–73.

3. Albano D, Bertagna F, Bertolia M, Bosio G, Lucchini S, Motta F, et al. INCIDENTAL FINDINGS SUGGESTIVE OF COVID-19 IN ASYMPTOMATIC PATIENTS UNDERGOING NUCLEAR MEDICINE PROCEDURES IN A HIGH PREVALENCE REGION. Journal of nuclear medicine : official publication, Society of Nuclear Medicine [Internet]. 2020; Available from: http://jnm.snmjournals.org/content/61/5/632.full.pdf+html

4. Albarello F, Pianura E, Di Stefano F, Cristofaro M, Petrone A, Marchioni L, et al. 2019-novel Coronavirus severe adult respiratory distress syndrome in two cases in Italy: An uncommon radiological presentation. International Journal of Infectious Diseases. 2020;93:192–7.

5. Alberici F, Del Barba E, Manenti C, Econimo L, Valerio F, Pola A, et al. Managing patients in dialysis and with kidney transplant infected with Covid-19. Giornale italiano di nefrologia : organo ufficiale della Societa italiana di nefrologia [Internet]. 2020;37(2). Available from: https://giornaleitalianodinefrologia.it/wp-content/uploads/sites/3/2020/04/37-02-2020-2.pdf

6. Alberici F, Delbarba E, Manenti C, Econimo L, Valerio F, Pola A, et al. Management Of Patients On Dialysis And With Kidney Transplant During SARS-COV-2 (COVID-19) Pandemic In Brescia, Italy. Kidney International Reports. 2020 Apr;

7. Amparore D, Claps F, Cacciamani GE, Esperto F, Fiori C, Liguori G, et al. Impact of the COVID-19 pandemic on urology residency training in Italy. Minerva urologica e nefrologica = The Italian journal of urology and nephrology [Internet]. 2020; Available from: https://www.researchgate.net/profile/Riccardo_Campi/publication/340511771_Impact_of_the_COVID-19_pandemic_on_urology_residency_training_in_Italy/links/5e91adeaa6fdcca7890a59e7/Impact-of-the-COVID-19-pandemic-on-urology-residency-training-in-Italy.pdf

8. Anelli F, Leoni G, Monaco R, Nume C, Rossi RC, Marinoni G, et al. Italian doctors call for protecting healthcare workers and boosting community surveillance during covid-19 outbreak. The BMJ [Internet]. 2020;368. Available from: https://www.bmj.com/content/368/bmj.m1254.full

9. Angelico R, Trapani S, Manzia TM, Lombardini L, Tisone G, Cardillo M. The COVID-19 outbreak in Italy: initial implications for organ transplantation programs. American journal of transplantation : official journal of the American Society of Transplantation and the American Society of Transplant Surgeons [Internet]. 2020; Available from: https://onlinelibrary.wiley.com/doi/full/10.1111/ajt.15904?casa_token=g74_lFOEN84AAAAA%3A3_W7nUmLiLq7cJAZHq9IGvi9zVx9XhyjUWRq6w3mKdCRzfl-iay7HpmfX94N5uIAoMcN3KzM-7uXnng

10. Antonelli G, Capobianchi MR, Riva E. The SARS-CoV-2 epidemic: how the Italian public is being informed. Clinical Microbiology and Infection: The Official Publication of the European Society of Clinical Microbiology and Infectious Diseases. 2020 Apr;

11. Arduino PG, Conrotto D, Broccoletti R. The outbreak of Novel Coronavirus disease (COVID-19) caused a worrying delay in the diagnosis of oral cancer in north-west Italy: the Turin Metropolitan Area experience. Oral Diseases. 2020 Apr;

12. Armocida B, Formenti B, Ussai S, Palestra F, Missoni E. The Italian health system and the COVID-19 challenge. The Lancet Public Health [Internet]. 2020; Available from: https://www.thelancet.com/journals/lanpub/article/PIIS2468-2667(20)30074-8/fulltext

13. Asperges E, Novati S, Muzzi A, Biscarini S, Sciarra M, Lupi M, et al. Rapid response to COVID-19 outbreak in Northern Italy: how to convert a classic infectious disease ward into a COVID-19 response centre. The Journal of Hospital Infection. 2020 Mar;

14. Atzori L, Mugheddu C, Addis G, Sanna S, Satta R, Ferreli C, et al. Psoriasis health care in the time of the coronavirus pandemic: insights from dedicated centers in sardinia (Italy). Journal of the European Academy of Dermatology and Venereology: JEADV. 2020 Apr;

15. Balduzzi A, Brivio E, Rovelli A, Rizzari C, Gasperini S, Melzi ML, et al. Lessons after the early management of the COVID-19 outbreak in a pediatric transplant and hemato-oncology center embedded within a COVID-19 dedicated hospital in Lombardia, Italy. Estote parati. Bone Marrow Transplantation. 2020 Apr;

16. Baracchini C, Pieroni A, Viaro F, Cianci V, Cattelan AM, Tiberio I, et al. Acute stroke management pathway during Coronavirus-19 pandemic. Neurological sciences : official journal of the Italian Neurological Society and of the Italian Society of Clinical Neurophysiology [Internet]. 2020; Available from: https://idp.springer.com/authorize/casa?redirect_uri=https://link.springer.com/content/pdf/10.1007/s10072-020-04375-9.pdf&casa_token=wNcUvd8NALoAAAAA:-SmoX2lUyus5vm2OhPM0AwFI0U9qKSdYXF3_EVKkb9kRwfVMVimo_I5fyX5tCrzMkTP1zRbBctAeWteu

17. Barone-Adesi F, Ragazzoni L, Schmid M. Investigating the determinants of high Case-Fatality Rate for COVID-19 in Italy. Disaster Medicine and Public Health Preparedness. 2020 Apr;1–4.

18. Bernucci C, Brembilla C, Veiceschi P. Effects of the COVID-19 Outbreak in Northern Italy: Perspectives from the Bergamo Neurosurgery Department. World Neurosurgery

[Internet]. 2020; Available from: https://www.wfns.org/WFNSData/Uploads/files/Effects-of-the-COVID-19-Outbreak-in-Northern-Italy-Perspectives-from-the-Bergamo-Neurosurgery-Department.pdf

19. Bersano A, Pantoni L. On being a neurologist in Italy at the time of the COVID-19 outbreak. Neurology. 2020 Apr;

20. Bhoori S, Rossi RE, Citterio D, Mazzaferro V. COVID-19 in long-term liver transplant patients: preliminary experience from an Italian transplant centre in Lombardy. The Lancet Gastroenterology and Hepatology [Internet]. 2020; Available from: https://www.ncbi.nlm.nih.gov/pmc/articles/PMC7146678/

21. Bianconi A, Marcelli A, Campi G, Perali A. Ostwald growth rate in controlled covid-19 epidemic spreading as in arrested growth in quantum complex matter. Condensed Matter [Internet]. 2020;5(2). Available from: https://www.mdpi.com/2410-3896/5/2/23/htm

22. Boccia S, Ricciardi W, Ioannidis JPA. What Other Countries Can Learn from Italy during the COVID-19 Pandemic. JAMA Internal Medicine [Internet]. 2020; Available from: https://www.scopus.com/inward/record.uri?eid=2-s2.0-85083156340&doi=10.1001%2fjamainternmed.2020.1447&partnerID=40&md5=3438ea778cd7483c281df529214b5e17

23. Boldrini P, Bernetti A, Fiore P. Impact of COVID-19 outbreak on rehabilitation services and Physical and Rehabilitation Medicine (PRM) physicians' activities in Italy. An official document of the Italian PRM Society (SIMFER). European journal of physical and rehabilitation medicine [Internet]. 2020; Available from: https://www.minervamedica.it/en/getfreepdf/jX1gcuUKufDdL8GCfszwB5atibSD6pZ8R%252BUwf4zqBs0xtk1GBDIfqV%252FVBiDwej3ZZI6BRWpXGUt4Dse888gHRQ%253D%253D/R33Y9999N00A20031601.pdf

24. Boldrini P, Kiekens C, Bargellesi S, Brianti R, Galeri S, Lucca L, et al. First impact on services and their preparation. 'Instant paper from the field' on rehabilitation answers to the Covid-19 emergency. European journal of physical and rehabilitation medicine [Internet]. 2020; Available from: https://www.minervamedica.it/en/getfreepdf/PipqD68ZQVGL95E%252BQmwn3MydXqZcyHYCUeviCZG%252Bo2Dbk7UVMYjwPMdrUE53sxFUngk3tJavMQxVRloqcUOEUA%253D%253D/R33Y9999N00A20040801.pdf

25. Bonalumi G, di Mauro M, Garatti A, Barili F, Gerosa G, Parolari A, et al. The COVID-19 outbreak and its impact on hospitals in Italy: the model of cardiac surgery. European Journal of Cardio-Thoracic Surgery: Official Journal of the European Association for Cardio-Thoracic Surgery. 2020 Apr;

26. Bordi L, Nicastri E, Scorzolini L, Di Caro A, Capobianchi MR, Castilletti C, et al. Differential diagnosis of illness in patients under investigation for the novel coronavirus (SARS-CoV-2), Italy, February 2020. Eurosurveillance [Internet]. 2020;25(8). Available from: https://www.eurosurveillance.org/content/10.2807/1560-7917.ES.2020.25.8.2000170

27. Borghetti A, Ciccullo A, Visconti E, Tamburrini E, Di Giambenedetto S. COVID-19 diagnosis does not rule out other concomitant diseases. European Journal of Clinical Investigation. 2020 Apr;e13241.

28. Borrelli E, Sacconi R, Querques L, Zucchiatti I, Prascina F, Bandello F, et al. Taking the right measures to control COVID-19 in ophthalmology: the experience of a tertiary eye care referral center in Italy. Eye (London, England). 2020 Apr;

29. Brioni E, Leopaldi D, Magnaghi C, Franchetti R, Granellini E, Pegoraro M, et al. Covid-19 in patients on dialysis: infection prevention and control strategies. Giornale italiano di nefrologia : organo ufficiale della Societa italiana di nefrologia [Internet]. 2020;37(2). Available from: https://giornaleitalianodinefrologia.it/wp-content/uploads/sites/3/2020/04/37-02-2020-5.pdf

30. Buonsenso D, Piano A, Raffaelli F, Bonadia N, de Gaetano Donati K, Franceschi F. Point-of-Care Lung Ultrasound findings in novel coronavirus disease-19 pnemoniae: A case report and potential applications during COVID-19 outbreak. European Review for Medical and Pharmacological Sciences. 2020;24(5):2776–80.

31. Caccialanza R, Laviano A, Lobascio F, Montagna E, Bruno R, Ludovisi S, et al. Early nutritional supplementation in non-critically ill patients hospitalized for the 2019 novel coronavirus disease (COVID-19): Rationale and feasibility of a shared pragmatic protocol. Nutrition [Internet]. 2020; Available from: https://www.sciencedirect.com/science/article/pii/S0899900720301180?casa_token=l5Liivll_doAAAAA:gR8xz97JgfYvad94HZ2OdXDccBZylF686RTiXJ-YwOgKnCgDdFs0BWfwKPlRzLi6jdx247b9oA

32. Campi R, Amparore D, Capitanio U, Checcucci E, Salonia A, Fiori C, et al. Assessing the Burden of Nondeferrable Major Uro-oncologic Surgery to Guide Prioritisation Strategies During the COVID-19 Pandemic: Insights from Three Italian High-volume Referral Centres. European Urology [Internet]. 2020; Available from: https://www.sciencedirect.com/science/article/pii/S0302283820302293?casa_token=k4gGU3eu6uoAAAAA:nD7QBuaheg-2XV1y2iLuFFyqyw4CWLmcboVnanNznlt8z4R-LOEpHk47031mEvH_TCQ6ZqYFiQ

33. Capobianchi MR, Rueca M, Messina F, Giombini E, Carletti F, Colavita F, et al. Molecular characterization of SARS-CoV-2 from the first case of COVID-19 in Italy. Clinical microbiology and infection : the official publication of the European Society of Clinical Microbiology and Infectious Diseases [Internet]. 2020; Available from: https://www.ncbi.nlm.nih.gov/pmc/articles/PMC7118617/

34. Carenzo L, Costantini E, Greco M, Barra FL, Rendiniello V, Mainetti M, et al. Hospital surge capacity in a tertiary emergency referral centre during the COVID-19 outbreak in Italy. Anaesthesia. 2020 Apr;

35. Carinci F. Covid-19: Preparedness, decentralisation, and the hunt for patient zero. The BMJ [Internet]. 2020;368. Available from: https://www.bmj.com/content/368/bmj.m799

36. Carletti F, Lalle E, Messina F, Ippolito G, Capobianchi MR. About the origin of the first two Sars-CoV-2 infections in Italy: inference not supported by appropriate sequence analysis. Journal of medical virology [Internet]. 2020; Available from: https://onlinelibrary.wiley.com/doi/epdf/10.1002/jmv.25833

37. Caruso D, Zerunian M, Polici M, Pucciarelli F, Polidori T, Rucci C, et al. Chest CT Features of COVID-19 in Rome, Italy. Radiology. 2020 Apr;201237.

38. Casanova M, Bagliacca EP, Silva M, Patriarca C, Veneroni L, Clerici CA, et al. How young patients with cancer perceive the Covid-19 (coronavirus) epidemic in Milan, Italy: is there room for other fears? Pediatric blood & cancer. 2020;e28318.

39. Cascella M, Rajnik M, Cuomo A, Dulebohn SC, Di Napoli R. Features, Evaluation and Treatment Coronavirus (COVID-19). In: StatPearls [Internet]. Treasure Island (FL): StatPearls Publishing; 2020. Available from: http://www.ncbi.nlm.nih.gov/books/NBK554776/

40. Cenzato M, DiMeco F, Fontanella M, Locatelli D, Servadei F. Editorial. Neurosurgery in the storm of COVID-19: suggestions from the Lombardy region, Italy (ex malo bonum). Journal of Neurosurgery. 2020 Apr;1–2.

41. Chintalapudi N, Battineni G, Amenta F. COVID-19 virus outbreak forecasting of registered and recovered cases after sixty day lockdown in Italy: A data driven model approach. Journal of Microbiology, Immunology and Infection [Internet]. 2020; Available from: https://www.sciencedirect.com/science/article/pii/S1684118220300980

42. Chirico F, Nucera G, Magnavita N. COVID-19: Protecting Healthcare Workers is a priority. Infection Control and Hospital Epidemiology. 2020 Apr;1–4.

43. Chirumbolo S. Might the many positive COVID19 subjects in Italy have been caused by resident bat-derived zoonotic β-coronaviruses instead of the Wuhan (China) outbreak? Journal of Medical Virology [Internet]. 2020; Available from: https://www.ncbi.nlm.nih.gov/pmc/articles/PMC7228365/

44. Chisci E, Masciello F, Michelagnoli S. Creation of a vascular surgical Hub responding to the COVID-19 emergency: the italian USL Toscana Centro model. Journal of Vascular Surgery. 2020 Apr;

45. Ciavattini A, Delli Carpini G, Giannella L, De Vincenzo R, Frega A, Cattani P, et al. Expert consensus from the Italian Society for Colposcopy and Cervico-Vaginal Pathology (SICPCV) for colposcopy and outpatient surgery of the lower genital tract during the COVID-19 pandemic. International journal of gynaecology and obstetrics: the official organ of the International Federation of Gynaecology and Obstetrics [Internet]. 2020; Available from: https://obgyn.onlinelibrary.wiley.com/doi/abs/10.1002/ijgo.13158?casa_token=_CfonOTcdF4AAAAA:LBPN1XwMcQbmgVKpTmF3QcA9X5AyZXB6cdKxe7_fqMBvEpYT0K_ego5KSV_VSboRxjSlR5N13DVHc-o

46. Ciccozzi M, Giovanetti M, Benvenuto D, Angeletti S. Response to Carletti et al, “About the origin of the first two SARS-CoV-2 infections in Italy: Inference not supported by appropriate sequence analysis”. Journal of Medical Virology [Internet]. 2020; Available from: https://onlinelibrary.wiley.com/doi/abs/10.1002/jmv.25823

47. Cioffi A. Professional autonomy and liability of the resident doctor: Between the hammer and the anvil. Journal of Forensic and Legal Medicine [Internet]. 2020;72. Available from: https://www.sciencedirect.com/science/article/pii/S1752928X2030072X?casa_token=RruPJoJcMQQAAAAA:Xr0PW8sf3jYnWdUIFaGZbYfD1dBOFoByGIxtXuhbcMjLoCyn5pCLGC5mFYRSDrHdxrhM_7aI_g

48. Ciufolini I, Paolozzi A. Mathematical prediction of the time evolution of the COVID-19 pandemic in Italy by a Gauss error function and Monte Carlo simulations. European Physical Journal Plus. 2020;135(4):355.

49. Cocci A, Presicce F, Russo GI, Cacciamani G, Cimino S, Minervini A. How sexual medicine is facing the outbreak of COVID-19: experience of Italian urological community and future perspectives. International Journal of Impotence Research. 2020 Apr;

50. Coccolini F, Perrone G, Chiarugi M, Di Marzo F, Ansaloni L, Scandroglio I, et al. Surgery in COVID-19 patients: Operational directives. World Journal of Emergency Surgery [Internet]. 2020;15(1). Available from: https://link.springer.com/content/pdf/10.1186/s13017-020-00307-2.pdf

51. Colaneri M, Seminari E, Piralla A, Zuccaro V, Filippo AD, Baldanti F, et al. Lack of SARS-CoV-2 RNA environmental contamination in a tertiary referral hospital for infectious diseases in Northern Italy. The Journal of Hospital Infection. 2020 Mar;

52. Colavita F, Lapa D, Carletti F, Lalle E, Bordi L, Marsella P, et al. SARS-CoV-2 Isolation From Ocular Secretions of a Patient With COVID-19 in Italy With Prolonged Viral RNA Detection. Annals of Internal Medicine. 2020 Apr;

53. Conticini E, Frediani B, Caro D. Can atmospheric pollution be considered a co-factor in extremely high level of SARS-CoV-2 lethality in Northern Italy? Environmental Pollution [Internet]. 2020; Available from: https://www.sciencedirect.com/science/article/pii/S0269749120320601?casa_token=sHejgBRbqx0AAAAA:t27Qj4gmeqTMNTglKZ2GsFA0Eb2731mxVQdX30WPZTfbjbT7549WhgFh-vWdZgASvO0i-ZwWKg

54. Contini C, Nuzzo MD, Barp N, Bonazza A, de Giorgio R, Tognon M, et al. The novel zoonotic COVID-19 pandemic: An expected global health concern. Journal of Infection in Developing Countries. 2020;14(3):254–64.

55. Corsini A, Bisciotti GN, Eirale C, Volpi P. Football cannot restart soon during the COVID-19 emergency! A critical perspective from the Italian experience and a call for action. British Journal of Sports Medicine [Internet]. 2020; Available from: https://bjsm.bmj.com/content/early/2020/05/23/bjsports-2020-102306.full

56. Cossarizza A, Gibellini L, De Biasi S, Lo Tartaro D, Mattioli M, Paolini A, et al. Handling and Processing of Blood Specimens from Patients with COVID-19 for Safe Studies on Cell Phenotype and Cytokine Storm. Cytometry Part A: The Journal of the International Society for Analytical Cytology. 2020 Apr;

57. Costi S, Caporali R, Cimaz R. Dealing with COVID-19 in a Pediatric Rheumatology Unit in Italy. Paediatric Drugs. 2020 Apr;

58. Curigliano G. How to Guarantee the Best of Care to Patients with Cancer During the COVID-19 Epidemic: The Italian Experience. The Oncologist. 2020 Apr;

59. D'Antiga L. Coronaviruses and immunosuppressed patients. The facts during the third epidemic. Liver transplantation : official publication of the American Association for the Study of Liver Diseases and the International Liver Transplantation Society [Internet]. 2020; Available from: https://aasldpubs.onlinelibrary.wiley.com/doi/abs/10.1002/lt.25756?casa_token=i0X3JYqMD54AAAAA:58cg-tAFHRNtzjZmVrkGRipJIiam5Lo8jOjqIrNnBQwjdhpR8vIm93ZfvlO0tcuah5hQn3V0Lkp_yx0

60. Danese S, Ran ZH, Repici A, Tong J, Omodei P, Aghemo A, et al. Gastroenterology department operational reorganisation at the time of covid-19 outbreak: an Italian and Chinese experience. Gut. 2020 Apr;

61. Dario Mandato V, Aguzzoli L. Management of ovarian cancer during the COVID-19 pandemic. International Journal of Gynaecology and Obstetrics: The Official Organ of the International Federation of Gynaecology and Obstetrics. 2020 Apr;

62. Davanzo R, Moro G, Sandri F, Agosti M, Moretti C, Mosca F. Breastfeeding and Coronavirus Disease-2019. Ad interim indications of the Italian Society of Neonatology endorsed by the Union of European Neonatal & Perinatal Societies. Maternal & child nutrition. 2020;e13010.

63. De Giorgio A. COVID-19 is not just a flu. Learn from Italy and act now. Travel Medicine and Infectious Disease. 2020 Apr;101655.

64. de Leo D, Trabucchi M. The Fight Against Covid-19: A Report From The Italian Trenches. International Psychogeriatrics. 2020 Apr;1–8.

65. De Santis L, Anastasi A, Cimadomo D, Klinger FG, Licata E, Pisaturo V, et al. COVID-19: the perspective of Italian embryologists managing the IVF laboratory in pandemic emergency. Human reproduction (Oxford, England) [Internet]. 2020; Available from: https://watermark.silverchair.com/deaa074.pdf?token=AQECAHi208BE49Ooan9kkhW_Ercy7Dm3ZL_9Cf3qfKAc485ysgAAAngwggJ0BgkqhkiG9w0BBwagggJlMIICYQIBADCCAloGCSqGSIb3DQEHATAeBglghkgBZQMEAS4wEQQMjOemLl1fljXzK5akAgEQgIICK8siea_UWJmwcAlD4kQCK-zZIfqmlUD4mRdMjtQPw0MaQBp61phmq26bEz4602zuYRWihpfEHCWYF91vFhl5a9BHVWEZRHAz6QBzbF_RHAsp2uSLc-tmNEaQIq9z1lcg5ovg9-

4Kw56Nn8br6KBukS9IlappkEgIxw1Ke3VQ3luQePrJNNUZ-BNCrKh7CQ3D5Wa4Hgimht0hfHsUCUapK6btuy0sI-0OiR9Xd7RZwARq4n-HjZTRT62UpdPQJdSmsM69DzFEIpKCrCJAt7gtZOaJeHypl-yPFemsLmfry7MQ6CyGb9uoARpQAvra50kBZQJYIgoBxaUlkbFjlEp1aRw-6UTOai51t0QBrdHydXYS4bYnPuSUVQdFCM8qONH3Ihu_vlpEF6jhN7FydOeCtEN97C2EH0ZJVhi9piVFfMUWPu7-LiYY3wjphdzDjqc3tJ1Hj4ipT2LluBfx_dArth8x56s2n9gC0ItDJCcOoJNPkoUX-cpm4FdPAokNcXZ9_pNLOwWyN3A_sBPphXnOHOfkHC5wCgrr4PWPNrpaEMoyGleBjlVGJrPXduZ_g-rw0PQnb-KxvCP8FH-zstKfplCzItfB6MjPSa9JefoUQb9wAZyEk1L1ocsGJg-ClxwKvaAVEXslYioo1P0i0COf4cIsLWimPRO838DjtMy_AtuaWhwHuzPhyV4PqeMU2saQTFPwaYLlbw74By1XsXDE4XSvS4Z7y40sar10hQ

66. Del Buono MG, Iannaccone G, Camilli M, Del Buono R, Aspromonte N. The Italian Outbreak of COVID-19: Conditions, Contributors, and Concerns. Mayo Clinic Proceedings [Internet]. 2020; Available from: https://www.researchgate.net/publication/340556811_The_Italian_Outbreak_of_COVID-19_Conditions_Contributors_and_Concerns

67. Department ICP, Morettini M, Sbrollini A, Marcantoni I, Burattini L. COVID-19 in Italy: Dataset of the Italian Civil Protection Department. Data in Brief [Internet]. 2020;30. Available from: https://www.sciencedirect.com/science/article/pii/S2352340920304200

68. Di Pasquale G. COVID-19 coronavirus: What implications for cardiology? [Coronavirus COVID-19: Quali implicazioni per la Cardiologia?]. Giornale Italiano di Cardiologia. 2020;21(4):243–5.

69. Di Saverio S, Pata F, Gallo G, Carrano F, Scorza A, Sileri P, et al. Coronavirus pandemic and Colorectal surgery: practical advice based on the Italian experience. Colorectal disease : the official journal of the Association of Coloproctology of Great Britain and Ireland [Internet]. 2020; Available from: https://onlinelibrary.wiley.com/doi/epdf/10.1111/codi.15056

70. Di Saverio S, Khan M, Pata F, Ietto G, De Simone B, Zani E, et al. Laparoscopy at all costs? Not now during COVID-19 and not for acute care surgery and emergency colorectal surgery: a practical algorithm from a Hub Tertiary teaching hospital in Northern Lombardy, Italy. The Journal of Trauma and Acute Care Surgery. 2020 Apr;

71. Dobran M, Paracino R, Iacoangeli M. Letter to the editor by Dobran Mauro, Paracino Riccardo, and Iacoangeli Maurizio regarding ‘Neurosurgery during the COVID-19 pandemic: update from Lombardy, northern Italy.’ Acta Neurochirurgica. 2020 Apr;

72. Duca A, Piva S, Focà E, Latronico N, Rizzi M. Calculated Decisions: Brescia-COVID Respiratory Severity Scale (BCRSS)/Algorithm. Emergency Medicine Practice. 2020 Apr;22(5 Suppl):CD1–2.

73. Faccincani R, Pascucci F, Lennquist S. How to surge to face SARS-CoV-2 outbreak. Lessons learned from Lumbardy, Italy. Disaster Medicine and Public Health Preparedness

[Internet]. 2020; Available from: https://www.cambridge.org/core/services/aop-cambridge-core/content/view/BC0C5DDF2280EBF6FA8C792E88965C0E/S1935789320000646a.pdf/how_to_surge_to_face_the_sarscov2_outbreak_lessons_learned_from_lombardy_italy.pdf

74. Fagiolini A, Cuomo A, Frank E. COVID-19 Diary From a Psychiatry Department in Italy. The Journal of clinical psychiatry [Internet]. 2020;81(3). Available from: https://ravikollimd.com/resources/COVID/Italy%201.pdf

75. Fanelli D, Piazza F. Analysis and forecast of COVID-19 spreading in China, Italy and France. Chaos, Solitons and Fractals [Internet]. 2020;134. Available from: https://www.sciencedirect.com/science/article/pii/S0960077920301636?casa_token=biz0d0XRpZoAAAAA:uQt7qYs3ifv_nUTbIYwjjEF0ZkMeebqKVsAbx7ziA-r53eLNy0JXWZKwJdAXwEww5A2EMtyO8A

76. Farina M, Barbisoni F, Bertacchini S, Borettaz I, Bucci R, Maggio M, et al. An account of the first hours of the Covid-19 epidemic at the Nephrology Unit in Lodi (Lombardy). Giornale italiano di nefrologia : organo ufficiale della Societa italiana di nefrologia [Internet]. 2020;37(2). Available from: https://giornaleitalianodinefrologia.it/en/tag/nephrology/

77. Ferrari D, Motta A, Strollo M, Banfi G, Locatelli M. Routine blood tests as a potential diagnostic tool for COVID-19. Clinical Chemistry and Laboratory Medicine. 2020 Apr;

78. Ferrazzi EM, Frigerio L, Cetin I, Vergani P, Spinillo A, Prefumo F, et al. COVID-19 Obstetrics Task Force, Lombardy, Italy: executive management summary and short report of outcome. International journal of gynaecology and obstetrics: the official organ of the International Federation of Gynaecology and Obstetrics [Internet]. 2020; Available from: https://obgyn.onlinelibrary.wiley.com/doi/full/10.1002/ijgo.13162?casa_token=CP6nWM9n1YkAAAAA%3AlMEnn_jau8TrSjyP7sjKve9P7jsJwnukbV5BlM0emjax0k4a2XsLOSwsZt56XOD0hcXUYOdKvKumxyM

79. Ficarra V, Novara G, Abrate A, Bartoletti R, Crestani A, De Nunzio C, et al. Urology practice during COVID-19 pandemic. Minerva urologica e nefrologica = The Italian journal of urology and nephrology [Internet]. 2020; Available from: https://www.minervamedica.it/en/getfreepdf/5TdwnQ64a9Nv6FnWv0MsXyZ8VXdZfRsiFpd0UMJ3AEnBvqgKet7u63jH%252FoBjIRi0pSDtAcjfabAOgTxYSC5aLA%253D%253D/R19Y9999N00A20032301.pdf

80. Filippi AR, Russi E, Magrini SM, Corvò R. Letter from Italy: First practical indications for radiation therapy departments during COVID-19 outbreak. International Journal of Radiation Oncology Biology Physics [Internet]. 2020; Available from: https://www.ncbi.nlm.nih.gov/pmc/articles/PMC7141469/pdf/main.pdf

81. Fineschi V, Aprile A, Aquila I, Arcangeli M, Asmundo A, Bacci M, et al. Management of the corpse with suspect, probable or confirmed COVID-19 respiratory infection - Italian interim recommendations for personnel potentially exposed to material from corpses,

including body fluids, in morgue structures and during autopsy practice. Pathologica. 2020 Mar;

82. Fiorino G, Colombo M, Natale C, Azzolini E, Lagioia M, Danese S. Clinician Education and Adoption of Preventive Measures for COVID-19: A Survey of a Convenience Sample of General Practitioners in Lombardy, Italy. Annals of Internal Medicine. 2020 Apr;

83. Franchini M, Farrugia A, Velati C, Zanetti A, Romanò L, Grazzini G, et al. The impact of the SARS-CoV-2 outbreak on the safety and availability of blood transfusions in Italy. Vox Sanguinis [Internet]. 2020; Available from: https://onlinelibrary.wiley.com/doi/pdf/10.1111/vox.12928?casa_token=wfkUiSFCW_UAAAAA:bBoxXuHM0La8dGsGJYYJW9KEutpMBXQ82ijaNKnILT0PM-Oj6ETpZpSsiLBjxDuALmCcf4n0CBytGzA

84. Franchini M, Marano G, Velati C, Pati I, Pupella S, Liumbruno GM. Operational protocol for donation of anti-COVID-19 convalescent plasma in Italy. Vox Sanguinis. 2020 Apr;

85. Gagliano A, Villani PG, Cò FM, Paglia S, Bisagni PAG, Perotti GM, et al. 2019-ncov's epidemic in middle province of northern Italy: Impact, logistic & strategy in the first line hospital. Disaster Medicine and Public Health Preparedness [Internet]. 2020; Available from: https://www.cambridge.org/core/services/aop-cambridge-core/content/view/AAEA381A70676F0A4505A23C2D006C62/S1935789320000518a.pdf/covid19_epidemic_in_the_middle_province_of_northern_italy_impact_logistics_and_strategy_in_the_first_line_hospital.pdf

86. Gallo G, La Torre M, Pietroletti R, Bianco F, Altomare DF, Pucciarelli S, et al. Italian society of colorectal surgery recommendations for good clinical practice in colorectal surgery during the novel coronavirus pandemic. Techniques in Coloproctology. 2020 Apr;

87. Gallo G, Trompetto M. The Effects of COVID-19 on Academic Activities and Surgical Education in Italy. Journal of Investigative Surgery [Internet]. 2020; Available from: https://covid19.elsevierpure.com/en/publications/the-effects-of-covid-19-on-academic-activities-and-surgical-educa

88. Gentile S, Strollo F, Ceriello A. COVID-19 infection in Italian people with diabetes: Lessons learned for our future (an experience to be used). Diabetes Research and Clinical Practice [Internet]. 2020;162. Available from: https://www.diabetesresearchclinicalpractice.com/action/showPdf?pii=S0168-8227%2820%2930387-9

89. Ghio S, Ferlini M, Scelsi L, Ferrario M, Camporotondo R, Vicentini A, et al. [COVID-19 pandemic: the need to reorganize a Cardiology Department in a hospital of the Lombardy Region, Italy]. Giornale Italiano Di Cardiologia (2006). 2020;21(5):358–9.

90. Giangreco G. Case fatality rate analysis of Italian COVID-19 outbreak. Journal of Medical Virology. 2020 Apr;

91. Gianotti R, Veraldi S, Recalcati S, Cusini M, Ghislanzoni M, Boggio F, et al. Cutaneous Clinico-Pathological Findings in three COVID-19-Positive Patients Observed in the Metropolitan Area of Milan, Italy. Acta Dermato-Venereologica [Internet]. 2020 Apr; Available from: https://www.ingentaconnect.com/contentone/mjl/adv/2020/00000100/f0030006/art00041?crawler=true&mimetype=application/pdf

92. Giordano G, Blanchini F, Bruno R, Colaneri P, Di Filippo A, Di Matteo A, et al. Modelling the COVID-19 epidemic and implementation of population-wide interventions in Italy. Nature Medicine [Internet]. 2020 Apr; Available from: https://www.nature.com/articles/s41591-020-0883-7.pdf

93. Giorgi PD, Villa F, Gallazzi E, Debernardi A, Schirò GR, Crisà FM, et al. The management of emergency spinal surgery during the COVID-19 pandemic in Italy. The Bone & Joint Journal. 2020 Apr;xxx.

94. Grasselli G, Pesenti A, Cecconi M. Critical Care Utilization for the COVID-19 Outbreak in Lombardy, Italy: Early Experience and Forecast during an Emergency Response. JAMA - Journal of the American Medical Association [Internet]. 2020; Available from: https://jamanetwork.com/journals/jama/article-abstract/2763188

95. Grasselli G, Zangrillo A, Zanella A, Antonelli M, Cabrini L, Castelli A, et al. Baseline Characteristics and Outcomes of 1591 Patients Infected with SARS-CoV-2 Admitted to ICUs of the Lombardy Region, Italy. JAMA - Journal of the American Medical Association [Internet]. 2020; Available from: https://www.scopus.com/inward/record.uri?eid=2-s2.0-85083171999&doi=10.1001%2fjama.2020.5394&partnerID=40&md5=0117ab53146aa98aefe682d017a8ccda

96. Guzzetta G, Poletti P, Ajelli M, Trentini F, Marziano V, Cereda D, et al. Potential short-term outcome of an uncontrolled COVID-19 epidemic in Lombardy, Italy, February to March 2020. Eurosurveillance [Internet]. 2020;25(12). Available from: https://www.eurosurveillance.org/content/10.2807/1560-7917.ES.2020.25.12.2000293?crawler=true&mimetype=application/pdf

97. Iaccarino G, Borghi C, Cicero AFG, Ferri C, Minuz P, Muiesan ML, et al. Renin-Angiotensin System Inhibition in Cardiovascular Patients at the Time of COVID19: Much Ado for Nothing? A Statement of Activity from the Directors of the Board and the Scientific Directors of the Italian Society of Hypertension. High Blood Pressure and Cardiovascular Prevention [Internet]. 2020; Available from: https://link.springer.com/content/pdf/10.1007/s40292-020-00380-3.pdf

98. Indini A, Aschele C, Cavanna L, Clerico M, Daniele B, Fiorentini G, et al. Reorganisation of medical oncology departments during the novel coronavirus disease-19 pandemic: a nationwide Italian survey. European Journal of Cancer. 2020;132:17–23.

99. Izzetti R, Nisi M, Gabriele M, Graziani F. COVID-19 Transmission in Dental Practice: Brief Review of Preventive Measures in Italy. Journal of Dental Research. 2020 Apr;22034520920580.

100. Kiekens C, Boldrini P, Andreoli A, Avesani R, Gamna F, Grandi M, et al. Rehabilitation and respiratory management in the acute and early post-acute phase. 'Instant paper from the field' on rehabilitation answers to the Covid-19 emergency. European Journal of Physical and Rehabilitation Medicine. 2020 Apr;

101. Krengli M, Ferrara E, Mastroleo F, Brambilla M, Ricardi U. Running a Radiation Oncology Department at the time of coronavirus: an Italian experience. Advances in Radiation Oncology. 2020 Mar;

102. Kurihara H, Bisagni P, Faccincani R, Zago M. COVID-19 OUTBREAK IN NORTHERN ITALY: VIEWPOINT OF THE MILAN AREA SURGICAL COMMUNITY. The Journal of Trauma and Acute Care Surgery [Internet]. 2020 Apr; Available from: https://sicut.net/wp-content/uploads/2020/04/COVID_19_OUTBREAK_IN_NORTHERN_ITALY__VIEWPOINT_OF.97966.pdf

103. La Maestra S, Abbondandolo A, De Flora S. Epidemiological trends of COVID-19 epidemic in Italy during March 2020. From 1,000 to 100,000 cases. Journal of Medical Virology. 2020 Apr;

104. La Manna G. More questions than answers, but a way ahead. Giornale italiano di nefrologia : organo ufficiale della Societa italiana di nefrologia [Internet]. 2020;37(2). Available from: https://www.scopus.com/inward/record.uri?eid=2-s2.0-85083319479&partnerID=40&md5=fb9f970769a2ee2bebe3d36bc5ed2834

105. La Marca A, Niederberger C, Pellicer A, Nelson SM. COVID-19: lessons from the Italian reproductive medical experience. Fertility and Sterility [Internet]. 2020; Available from: https://www.scopus.com/inward/record.uri?eid=2-s2.0-85082188750&doi=10.1016%2fj.fertnstert.2020.03.021&partnerID=40&md5=5adaaf58f0b4438f92896e76cb930089

106. Lambertini M, Toss A, Passaro A, Criscitiello C, Cremolini C, Cardone C, et al. Cancer care during the spread of coronavirus disease 2019 (COVID-19) in Italy: Young oncologists' perspective. ESMO Open [Internet]. 2020;5(2). Available from: https://www.scopus.com/inward/record.uri?eid=2-s2.0-85082730681&doi=10.1136%2fesmoopen-2020-000759&partnerID=40&md5=a13a4acba8b71d1e3943095ad50dbbdb

107. Lauro A, Pagano N, Impellizzeri G, Cervellera M, Tonini V. Emergency Endoscopy During the SARS-CoV-2 Pandemic in the North of Italy: Experience from St. Orsola University Hospital-Bologna. Digestive Diseases and Sciences. 2020 Apr;

108. Lazzeri M, Lanza A, Bellini R, Bellofiore A, Cecchetto S, Colombo A, et al. Respiratory physiotherapy in patients with COVID-19 infection in acute setting: A Position Paper of

the Italian Association of Respiratory Physiotherapists (ARIR). Monaldi Archives for Chest Disease. 2020;90(1):163–8.

109. Lazzerini M, Barbi E, Apicella A, Marchetti F, Cardinale F, Trobia G. Delayed access or provision of care in Italy resulting from fear of COVID-19. The Lancet Child and Adolescent Health [Internet]. 2020; Available from: https://www.scopus.com/inward/record.uri?eid=2-s2.0-85083336420&doi=10.1016%2fS2352-4642%2820%2930108-5&partnerID=40&md5=3cdf81f3aa42d1ae4979a478da158628

110. Lazzerini M, Putoto G. COVID-19 in Italy: momentous decisions and many uncertainties. The Lancet Global Health [Internet]. 2020; Available from: https://www.scopus.com/inward/record.uri?eid=2-s2.0-85082417744&doi=10.1016%2fS2214-109X%2820%2930110-8&partnerID=40&md5=a3d1e7dcc774eb3d1a9c3910eb9e7a75

111. Lenti MV, Corazza GR, Di Sabatino A. Carving out a place for internal medicine during COVID-19 epidemic in Italy. Journal of Internal Medicine. 2020 Apr;

112. Leva E, Morandi A, Sartori A, Macchini F, Berrettini A, Manzoni G. Correspondence from Northern Italy about our experience with COVID-19. Journal of Pediatric Surgery [Internet]. 2020; Available from: https://www.scopus.com/inward/record.uri?eid=2-s2.0-85083222374&doi=10.1016%2fj.jpedsurg.2020.03.028&partnerID=40&md5=1408dddd0bbab7270602b8f1122ef22d

113. Licastro D, Rajasekharan S, Dal Monego S, Segat L, D'Agaro P, Marcello A. Isolation and full-length genome characterization of SARS-CoV-2 from COVID-19 cases in Northern Italy. Journal of virology [Internet]. 2020; Available from: https://www.scopus.com/inward/record.uri?eid=2-s2.0-85083153612&doi=10.1128%2fJVI.00543-20&partnerID=40&md5=d0e6d76657613b1b3513af5d0d420f3d

114. Licciardi F, Giani T, Baldini L, Favalli EG, Caporali R, Cimaz R. COVID-19 and what pediatric rheumatologists should know: a review from a highly affected country. Pediatric Rheumatology Online Journal. 2020 Apr;18(1):35.

115. Lippi G, Mattiuzzi C, Sanchis-Gomar F, Henry BM. Clinical and demographic characteristics of patients dying from COVID-19 in Italy versus China. Journal of Medical Virology. 2020 Apr;

116. Lisi G, Campanelli M, Spoletini D, Carlini M. The possible impact of COVID-19 on colorectal surgery in Italy. Colorectal disease : the official journal of the Association of Coloproctology of Great Britain and Ireland [Internet]. 2020; Available from: https://www.scopus.com/inward/record.uri?eid=2-s2.0-85083274449&doi=10.1111%2fcodi.15054&partnerID=40&md5=85c271f41b48b3a3d8756486b0668e70

117. Lombardy Section Italian Society Infectious And Tropical Diseases -. Vademecum for the treatment of people with COVID-19. Edition 2.0, 13 March 2020. Le Infezioni in Medicina. 2020 Jun;28(2):143–52.

118. Lomoro P, Verde F, Zerboni F, Simonetti I, Borghi C, Fachinetti C, et al. COVID-19 pneumonia manifestations at the admission on chest ultrasound, radiographs, and CT: single-center study and comprehensive radiologic literature review. European Journal of Radiology Open [Internet]. 2020;7. Available from: https://www.scopus.com/inward/record.uri?eid=2-s2.0-85082831277&doi=10.1016%2fj.ejro.2020.100231&partnerID=40&md5=76ee94c7b14938ef596cab16a7e05e53

119. Lorusso A, Calistri P, Mercante MT, Monaco F, Portanti O, Marcacci M, et al. A 'One-Health' approach for diagnosis and molecular characterization of SARS-CoV-2 in Italy. One Health (Amsterdam, Netherlands). 2020 Apr;100135.

120. Luciani LG, Mattevi D, Cai T, Giusti G, Proietti S, Malossini G. Teleurology in the Time of Covid-19 Pandemic: Here to Stay? Urology [Internet]. 2020; Available from: https://www.scopus.com/inward/record.uri?eid=2-s2.0-85083329467&doi=10.1016%2fj.urology.2020.04.004&partnerID=40&md5=1c2d6a70f04f59373d3ceb8da16636a4

121. Malipiero G, Paoletti G, Puggioni F, Racca F, Ferri S, Marsala A, et al. An academic allergy unit during COVID-19 pandemic in Italy. The Journal of Allergy and Clinical Immunology. 2020 Apr;

122. Manganaro M, Baldovino S, Working group of the Piedmont and Aosta Valley Section of the SIN. First considerations on the SARS-CoV-2 epidemic in the Dialysis Units of Piedmont and Aosta Valley, Northern Italy. Journal of Nephrology. 2020 Apr;

123. Mannelli C. Whose life to save? Scarce resources allocation in the COVID-19 outbreak. Journal of Medical Ethics. 2020 Apr;

124. Marano L, Marrelli D, Roviello F. Cancer care under the outbreak of COVID-19: A perspective from Italian tertiary referral center for surgical oncology. European Journal of Surgical Oncology: The Journal of the European Society of Surgical Oncology and the British Association of Surgical Oncology. 2020 Apr;

125. Marasca C, Ruggiero A, Annunziata MC, Fabbrocini G, Megna M. Face the COVID-19 emergency: measures applied in an Italian Dermatologic Clinic. Journal of the European Academy of Dermatology and Venereology: JEADV. 2020 Apr;

126. Marietta M, Ageno W, Artoni A, De Candia E, Gresele P, Marchetti M, et al. COVID-19 and haemostasis: a position paper from Italian Society on Thrombosis and Haemostasis (SISET). Blood Transfusion = Trasfusione Del Sangue. 2020 Apr;

127. Martini N, Piccinni C, Pedrini A, Maggioni A. [CoViD-19 and chronic diseases: current knowledge, future steps and the MaCroScopio project.]. Recenti Progressi in Medicina. 2020;111(4):198–201.

128. Mascaretti L, De Angelis V, Berti P. The severe acute respiratory syndrome coronavirus 2 (SARS-CoV-2) pandemic and Transfusion Medicine: reflections from Italy. Blood transfusion = Trasfusione del sangue. 2020;18(2):77–8.

129. Mauri T, Spinelli E, Scotti E, Colussi G, Basile MC, Crotti S, et al. Potential for Lung Recruitment and Ventilation-Perfusion Mismatch in Patients With the Acute Respiratory Distress Syndrome From Coronavirus Disease 2019. Critical Care Medicine. 2020 Apr;

130. Meattini I, Franco P, Belgioia L, Boldrini L, Botticella A, De Santis MC, et al. Radiation therapy during the coronavirus disease 2019 (covid-19) pandemic in Italy: a view of the nation's young oncologists. ESMO open. 2020;5(2).

131. Miani A, Burgio E, Piscitelli P, Lauro R, Colao A. The Italian war-like measures to fight coronavirus spreading: Re-open closed hospitals now. EClinicalMedicine [Internet]. 2020; Available from: https://www.scopus.com/inward/record.uri?eid=2-s2.0-85082418705&doi=10.1016%2fj.eclinm.2020.100320&partnerID=40&md5=2ad0e0ac7f1b223264b1ed6b240b4613

132. Moccia L, Janiri D, Pepe M, Dattoli L, Molinaro M, De Martin V, et al. Affective temperament, attachment style, and the psychological impact of the COVID-19 outbreak: an early report on the Italian general population. Brain, Behavior, and Immunity. 2020 Apr;

133. Montorsi F. We Should Not Ignore What Scientific Articles are Telling US: A Lesson from the Italian COVID-19 Experience. The Journal of Urology. 2020 Apr;101097JU0000000000001069.

134. Moro M, Vigezzi GP, Capraro M, Biancardi A, Nizzero P, Signorelli C, et al. 2019-novel coronavirus survey: knowledge and attitudes of hospital staff of a large Italian teaching hospital. Acta Bio-Medica: Atenei Parmensis. 2020;91(3-S):29–34.

135. Motta I, De Amicis MM, Pinto VM, Balocco M, Longo F, Bonetti F, et al. SARS-CoV-2 infection in beta thalassemia: preliminary data from the Italian experience. American Journal of Hematology. 2020 Apr;

136. Mugheddu C, Dell'Antonia M, Sanna S, Agosta D, Atzori L, Rongioletti F. Successful Guselkumab treatment in a psoriatic patient affected with Cornelia de Lange Syndrome, and prosecution during the COVID-19 pandemic. Dermatologic Therapy. 2020 Apr;

137. Nicastri E, D'Abramo A, Faggioni G, De Santis R, Mariano A, Lepore L, et al. Coronavirus disease (COVID-19) in a paucisymptomatic patient: Epidemiological and clinical challenge in settings with limited community transmission, Italy, February 2020. Eurosurveillance [Internet]. 2020;25(11). Available from: https://www.scopus.com/inward/record.uri?eid=2-s2.0-

85082380423&doi=10.2807%2f1560-7917.ES.2020.25.11.2000230&partnerID=40&md5=783bb305916c3c6e23de7513775ec558

138. Nicastri E, Petrosillo N, Bartoli TA, Lepore L, Mondi A, Palmieri F, et al. National Institute for the Infectious Diseases ‘L. Spallanzani’, IRCCS. Recommendations for COVID-19 clinical management. Infectious Disease Reports. 2020 Feb;12(1):8543.

139. Nicoli F, Gasparetto A. Italy in a Time of Emergency and Scarce Resources: The Need for Embedding Ethical Reflection in Social and Clinical Settings. The Journal of clinical ethics. 2020;31(1):92–4.

140. Norsa L, Indriolo A, Sansotta N, Cosimo P, Greco S, D’Antiga L. Uneventful course in IBD patients during SARS-CoV-2 outbreak in northern Italy. Gastroenterology. 2020 Apr;

141. Occhipinti V, Pastorelli L. Challenges in the Care of IBD Patients During the CoViD-19 Pandemic: Report From a ‘Red Zone’ Area in Northern Italy. Inflammatory Bowel Diseases. 2020 Apr;

142. Omboni S. Telemedicine During The COVID-19 in Italy: A Missed Opportunity? Telemedicine Journal and E-Health: The Official Journal of the American Telemedicine Association. 2020 Apr;

143. Onder G, Rezza G, Brusaferro S. Case-Fatality Rate and Characteristics of Patients Dying in Relation to COVID-19 in Italy. JAMA - Journal of the American Medical Association [Internet]. 2020; Available from: https://www.scopus.com/inward/record.uri?eid=2-s2.0-85082315325&doi=10.1001%2fjama.2020.4683&partnerID=40&md5=b106e4b9611d85041a2e165e9025c479

144. Pachetti M, Marini B, Benedetti F, Giudici F, Mauro E, Storici P, et al. Emerging SARS-CoV-2 mutation hot spots include a novel RNA-dependent-RNA polymerase variant. Journal of Translational Medicine. 2020 Apr;18(1):179.

145. Pacifico A, Ardigò M, Frascione P, Damiani G, Morrone A. Phototherapeutic approach to dermatological patients during the 2019 Coronavirus pandemic: Real-life Data from the Italian Red Zone. The British Journal of Dermatology. 2020 Apr;

146. Pagnini C, Urgesi R, Di Paolo MC, Graziani MG. Fighting the battle against SARS-CoV-2 as gastroenterologists in Italy. Gastroenterology. 2020 Apr;

147. Pedersini P, Corbellini C, Villafañe JH. Italian Physical Therapists’ Response to the Novel COVID-19 Emergency. Physical Therapy. 2020 Apr;

148. Pellino G, Spinelli A. How COVID-19 Outbreak Is Impacting Colorectal Cancer Patients in Italy: A Long Shadow Beyond Infection. Diseases of the colon and rectum [Internet]. 2020; Available from: https://www.scopus.com/inward/record.uri?eid=2-s2.0-85082695055&doi=10.1097%2fDCR.0000000000001685&partnerID=40&md5=3ed7607a5c6e4e8017195328b0c4a3ec

149. Percudani M, Corradin M, Moreno M, Indelicato A, Vita A. Mental Health Services in Lombardy during COVID-19 outbreak. Psychiatry Research [Internet]. 2020;288. Available from: https://www.scopus.com/inward/record.uri?eid=2-s2.0-85083343089&doi=10.1016%2fj.psychres.2020.112980&partnerID=40&md5=e20133adbecf99117df96af36891a20d

150. Piccinni M, Aprile A, Benciolini P, Busatta L, Cadamuro E, Malacarne P, et al. [Ethical, deontologic and legal considerations about SIAARTI Document 'Clinical ethics recommendations for the allocation of intensive care treatments, in exceptional, resource-limited circumstances'.]. Recenti Progressi in Medicina. 2020 Apr;111(4):212–22.

151. Piepoli MF, Emdin M. A dialogue between the editor-in-chief and a deputy editor of a cardiology journal during the coronavirus outbreak: Take-home messages from the Italian experience. European Journal of Preventive Cardiology [Internet]. 2020; Available from: https://www.scopus.com/inward/record.uri?eid=2-s2.0-85082923145&doi=10.1177%2f2047487320918077&partnerID=40&md5=6025bad860ec699f732c872097a417b7

152. Pietrantonio F, Garassino MC. Caring for Patients with Cancer during the COVID-19 Outbreak in Italy. JAMA Oncology [Internet]. 2020; Available from: https://www.scopus.com/inward/record.uri?eid=2-s2.0-85083243697&doi=10.1001%2fjamaoncol.2020.1426&partnerID=40&md5=74489ca7c546a10f9997d21ad642b7b3

153. Porcheddu R, Serra C, Kelvin D, Kelvin N, Rubino S. Similarity in Case Fatality Rates (CFR) of COVID-19/SARS-COV-2 in Italy and China. Journal of Infection in Developing Countries. 2020;14(2):125–8.

154. Porpiglia F, Checcucci E, Amparore D, Verri P, Campi R, Claps F, et al. Slowdown of urology residents' learning curve during COVID-19 emergency. BJU international. 2020 Apr;

155. Portaluri M, Tramacere F, Portaluri T, Gianicolo EAL. Southern Italy: How the supply of radiation therapy, patient outcomes, and risk to health care providers have changed during the COVID-19 Pandemic. Advances in Radiation Oncology. 2020 Apr;

156. Porzio G, Cortellini A, Bruera E, Verna L, Ravoni G, Peris F, et al. Home Care for Cancer Patients During COVID-19 Pandemic: The Double Triage Protocol. Journal of Pain and Symptom Management [Internet]. 2020; Available from: https://www.scopus.com/inward/record.uri?eid=2-s2.0-85083312698&doi=10.1016%2fj.jpainsymman.2020.03.021&partnerID=40&md5=527dc8628ca7a030cddf5d610e25cc98

157. Prati C, Pelliccioni GA, Sambri V, Chersoni S, Gandolfi MG. COVID-19: its impact on dental schools in Italy, clinical problems in endodontic therapy and general considerations. International endodontic journal. 2020;53(5):723–5.

158. Pulvirenti F, Cinetto F, Milito C, Bonanni L, Pesce AM, Leodori G, et al. Health-Related Quality of Life in Common Variable Immunodeficiency Italian Patients Switched to Remote Assistance During the COVID-19 Pandemic. Journal of Allergy and Clinical Immunology: In Practice [Internet]. 2020; Available from: https://www.scopus.com/inward/record.uri?eid=2-s2.0-85083306665&doi=10.1016%2fj.jaip.2020.04.003&partnerID=40&md5=e2ea91fdc535b787344523b2963684b7

159. Radi G, Diotallevi F, Campanati A, Offidani A. Global coronavirus pandemic (2019-nCOV): Implication for an Italian medium size dermatological clinic of a ii level hospital. Journal of the European Academy of Dermatology and Venereology : JEADV [Internet]. 2020; Available from: https://www.scopus.com/inward/record.uri?eid=2-s2.0-85083242511&doi=10.1111%2fjdv.16386&partnerID=40&md5=20110ea8aac3d14387fee821c02b08e1

160. Raffaetà R. Another Day in Dystopia. Italy in the Time of COVID-19. Medical Anthropology: Cross Cultural Studies in Health and Illness [Internet]. 2020; Available from: https://www.scopus.com/inward/record.uri?eid=2-s2.0-85082756152&doi=10.1080%2f01459740.2020.1746300&partnerID=40&md5=9eeb4e4b737ceb6a8cabebf5c444f271

161. Remuzzi A, Remuzzi G. COVID-19 and Italy: what next? The Lancet. 2020;395(10231):1225–8.

162. Repici A, Maselli R, Colombo M, Gabbiadini R, Spadaccini M, Anderloni A, et al. Coronavirus (COVID-19) outbreak: what the department of endoscopy should know. Gastrointestinal Endoscopy [Internet]. 2020; Available from: https://www.scopus.com/inward/record.uri?eid=2-s2.0-85082406228&doi=10.1016%2fj.gie.2020.03.019&partnerID=40&md5=5027a289c941013f71168efa85245943

163. Repici A, Pace F, Gabbiadini R, Colombo M, Hassan C, Dinelli M, et al. Endoscopy units and the COVID-19 Outbreak: A Multi-Center Experience from Italy. Gastroenterology. 2020 Apr;

164. Riccioni L, Bertolini G, Giannini A, Vergano M, Gristina G, Livigni S, et al. [Clinical ethics recommendations for the allocation of intensive care treatments, in exceptional, resource-limited circumstances.]. Recenti Progressi in Medicina. 2020 Apr;111(4):207–11.

165. Righi G, Del Popolo G. COVID-19 tsunami: the first case of a spinal cord injury patient in Italy. Spinal Cord Series and Cases. 2020;6(1):22.

166. Rizzi M, Castelli F, Latronico N, Focá E. SARS-CoV-2 invades the West. How to face a COVID-19 epidemic in Lombardy, Northern Italy? Le Infezioni in Medicina. 2020 Jun;28(2):133–4.

167. Rombolà G, Heidempergher M, Pedrini L, Farina M, Aucella F, Messa P, et al. Practical indications for the prevention and management of SARS-CoV-2 in ambulatory dialysis patients: lessons from the first phase of the epidemics in Lombardy. Journal of Nephrology. 2020;33(2):193–6.

168. Rossi ED, Fadda G, Mule A, Zannoni GF, Rindi G. Cytologic and histologic samples from patients infected by the novel coronavirus 2019 SARS-CoV-2: An italian institutional experience focusing on biosafety procedures. Cancer Cytopathology [Internet]. 2020; Available from: https://www.scopus.com/inward/record.uri?eid=2-s2.0-85082963146&doi=10.1002%2fcncy.22281&partnerID=40&md5=d7bc85d6dac264c413c4147195d42821

169. Rubino S, Kelvin N, Bermejo-Martin JF, Kelvin DJ. As COVID-19 cases, deaths and fatality rates surge in Italy, underlying causes require investigation. Journal of Infection in Developing Countries. 2020;14(3):265–7.

170. Saglietto A, D'Ascenzo F, Zoccai GB, De Ferrari GM. COVID-19 in Europe: the Italian lesson. The Lancet. 2020;395(10230):1110–1.

171. Saibene AM, Allevi F, Biglioli F, Felisati G. Role and Management of a Head and Neck Department during the COVID-19 Outbreak in Lombardy. Otolaryngology - Head and Neck Surgery (United States) [Internet]. 2020; Available from: https://www.scopus.com/inward/record.uri?eid=2-s2.0-85083061785&doi=10.1177%2f0194599820917914&partnerID=40&md5=53b3dd737b6853252545eb0b8ad11a50

172. Sainati L, Biffi A. How we deal with the COVID-19 epidemic in an Italian pediatric onco-hematology clinic located in a region at high density of cases. British Journal of Haematology. 2020 Apr;

173. Salvatori G, De Rose DU, Concato C, Alario D, Olivini N, Dotta A, et al. Managing COVID-19-Positive Maternal-Infant Dyads: An Italian Experience. Breastfeeding Medicine: The Official Journal of the Academy of Breastfeeding Medicine. 2020 Apr;

174. Sani G, Janiri D, Di Nicola M, Janiri L, Ferretti S, Chieffo D. Mental health during and after the COVID-19 emergency in Italy. Psychiatry and clinical neurosciences [Internet]. 2020; Available from: https://www.scopus.com/inward/record.uri?eid=2-s2.0-85083235253&doi=10.1111%2fpcn.13004&partnerID=40&md5=a572120fc09d4bf9aff7b11ec05a3a68

175. Santacroce L, Bottalico L, Charitos IA. The Impact of COVID-19 on Italy: A Lesson for the Future. The international journal of occupational and environmental medicine [Internet]. 2020; Available from: https://www.scopus.com/inward/record.uri?eid=2-s2.0-85083165375&doi=10.34172%2fijoem.2020.1984&partnerID=40&md5=b3840dec381cf72e7ed2fd14ed8cd849

176. Scarpioni R, Manini A, Valsania T, De Amicis S, Albertazzi V, Melfa L, et al. Covid-19 and its impact on nephropathic patients: the experience at Ospedale 'Guglielmo da

Saliceto’ in Piacenza. Giornale italiano di nefrologia : organo ufficiale della Societa italiana di nefrologia [Internet]. 2020;37(2). Available from: https://www.scopus.com/inward/record.uri?eid=2-s2.0-85083297732&partnerID=40&md5=6e497634ec7bac46b03f9aa6ee09d3d9

177. Sebastiani G, Massa M, Riboli E. Covid-19 epidemic in Italy: evolution, projections and impact of government measures. European Journal of Epidemiology. 2020 Apr;

178. Senni M. COVID-19 experience in Bergamo, Italy. European Heart Journal. 2020 Apr;

179. Signorelli C, Fara GM. COVID-19: Hygiene and Public Health to the front. Acta Bio-Medica: Atenei Parmensis. 2020;91(3-S):7–8.

180. Signorelli C, Scognamiglio T, Odone A. COVID-19 in Italy: impact of containment measures and prevalence estimates of infection in the general population. Acta Bio-Medica: Atenei Parmensis. 2020;91(3-S):175–9.

181. Silvestris N, Moschetta A, Paradiso A, Delvino A. COVID-19 Pandemic and the Crisis of Health Systems: The Experience of the Apulia Cancer Network and of the Comprehensive Cancer Center Istituto Tumori ‘Giovanni Paolo II’ of Bari. International Journal of Environmental Research and Public Health. 2020;17(8).

182. Simonato A, Giannarini G, Abrate A, Bartoletti R, Crestani A, De Nunzio C, et al. Pathways for urology patients during the COVID-19 pandemic. Minerva urologica e nefrologica = The Italian journal of urology and nephrology [Internet]. 2020; Available from: https://www.scopus.com/inward/record.uri?eid=2-s2.0-85083222740&doi=10.23736%2fS0393-2249.20.03861-8&partnerID=40&md5=2fee3a2cf461bf243f47125642cfe7f9

183. Sorbello M, El-Boghdadly K, Di Giacinto I, Cataldo R, Esposito C, Falcetta S, et al. The Italian coronavirus disease 2019 outbreak: recommendations from clinical practice. Anaesthesia [Internet]. 2020; Available from: https://www.scopus.com/inward/record.uri?eid=2-s2.0-85082436952&doi=10.1111%2fanae.15049&partnerID=40&md5=feeb1e781063bee7198eca52a5605d31

184. Soresina A, Moratto D, Chiarini M, Paolillo C, Baresi G, Focà E, et al. Two X-linked agammaglobulinemia patients develop pneumonia as COVID-19 manifestation but recover. Pediatric Allergy and Immunology: Official Publication of the European Society of Pediatric Allergy and Immunology. 2020 Apr;

185. Sotgiu G, Gerli GA, Centanni S, Miozzo M, Canonica GW, Soriano JB, et al. Advanced forecasting of SARS-CoV-2 related deaths in Italy, Germany, Spain, and New York State. Allergy. 2020 Apr;

186. Spagnuolo G, De Vito D, Rengo S, Tatullo M. COVID-19 outbreak: An overview on dentistry. International Journal of Environmental Research and Public Health [Internet]. 2020;17(6). Available from: https://www.scopus.com/inward/record.uri?eid=2-s2.0-

85082487208&doi=10.3390%2fijerph17062094&partnerID=40&md5=79afa80de4312cceb4f5d2eadffb341a

187. Spina S, Marrazzo F, Migliari M, Stucchi R, Sforza A, Fumagalli R. The response of Milan's Emergency Medical System to the COVID-19 outbreak in Italy. The Lancet. 2020;395(10227):e49–50.

188. Spinazzè A, Cattaneo A, Cavallo DM. COVID-19 outbreak in Italy: protecting worker health and the response of the Italian Industrial Hygienists Association. Annals of Work Exposures and Health. 2020 Apr;

189. Spinsanti S. [The cure: a question of merit?]. Recenti Progressi in Medicina. 2020;111(4):184–5.

190. Starace F, Ferrara M. COVID-19 disease Emergency Operational Instructions for Mental Health Departments issued by the Italian Society of Epidemiological Psychiatry. Epidemiology and psychiatric sciences. 2020;1–12.

191. Stefanelli P, Faggioni G, Lo Presti A, Fiore S, Marchi A, Benedetti E, et al. Whole genome and phylogenetic analysis of two SARSCoV-2 strains isolated in Italy in January and February 2020: Additional clues on multiple introductions and further circulation in Europe. Eurosurveillance [Internet]. 2020;25(13). Available from: https://www.scopus.com/inward/record.uri?eid=2-s2.0-85083038104&doi=10.2807%2f1560-7917.ES.2020.25.13.2000305&partnerID=40&md5=7354211cf28bdd9a9301f0b8475b9847

192. Stefanini GG, Azzolini E, Condorelli G. Critical Organizational Issues for Cardiologists in the COVID-19 Outbreak: A Frontline Experience From Milan, Italy. Circulation [Internet]. 2020; Available from: https://www.scopus.com/inward/record.uri?eid=2-s2.0-85083042975&doi=10.1161%2fCIRCULATIONAHA.120.047070&partnerID=40&md5=40f79115a7b0c90bb9353acd5dc14b98

193. Sverzellati N, Milone F, Balbi M. How imaging should properly be used in COVID-19 outbreak: an Italian experience. Diagnostic and interventional radiology (Ankara, Turkey) [Internet]. 2020; Available from: https://www.scopus.com/inward/record.uri?eid=2-s2.0-85083261830&doi=10.5152%2fdir.2020.30320&partnerID=40&md5=bb1e355a1256e00dafe3db2ee05a2cbd

194. Tarantini G, Fraccaro C, Chieffo A, Marchese A, Tarantino FF, Rigattieri S, et al. Italian Society of Interventional Cardiology (GISE) Position Paper for Cath lab-specific Preparedness Recommendations for Healthcare providers in case of suspected, probable or confirmed cases of COVID-19. Catheterization and cardiovascular interventions : official journal of the Society for Cardiac Angiography & Interventions [Internet]. 2020; Available from: https://www.scopus.com/inward/record.uri?eid=2-s2.0-85083159407&doi=10.1002%2fccd.28888&partnerID=40&md5=34b69e021c03387bf1648e5a7f0a5c47

195. Torretta S, Gaini LM, Pignataro L. Why Italian ENT physicians should be aware of SARS-CoV-2. Acta otorhinolaryngologica Italica : organo ufficiale della Societa italiana di otorinolaringologia e chirurgia cervico-facciale [Internet]. 2020; Available from: https://www.scopus.com/inward/record.uri?eid=2-s2.0-85083277817&doi=10.14639%2f0392-100X-N0738&partnerID=40&md5=4eecc033c7b6e6eb12818446fe4132d2

196. Tosato F, Giraudo C, Pelloso M, Musso G, Piva E, Plebani M. One disease, different features: COVID-19 laboratory and radiological findings in three Italian patients. Clinical chemistry and laboratory medicine [Internet]. 2020; Available from: https://www.scopus.com/inward/record.uri?eid=2-s2.0-85083194430&doi=10.1515%2fcclm-2020-0319&partnerID=40&md5=9034d0732f0e937566f06edd4501ca66

197. Vergano M, Bertolini G, Giannini A, Gristina GR, Livigni S, Mistraletti G, et al. Clinical ethics recommendations for the allocation of intensive care treatments in exceptional, resource-limited circumstances: the Italian perspective during the COVID-19 epidemic. Critical Care (London, England). 2020 Apr;24(1):165.

198. Vetrugno L, Bove T, Orso D, Barbariol F, Bassi F, Boero E, et al. Our Italian Experience Using Lung Ultrasound for Identification, Grading and Serial Follow-up of Severity of Lung Involvement for Management of Patients with COVID-19. Echocardiography (Mount Kisco, NY) [Internet]. 2020; Available from: https://www.scopus.com/inward/record.uri?eid=2-s2.0-85083258212&doi=10.1111%2fecho.14664&partnerID=40&md5=855c4896e8ab14b5296a6356fcc16b7a

199. Vigliar E, Iaccarino A, Bruzzese D, Malapelle U, Bellevicine C, Troncone G. Cytology in the time of coronavirus disease (covid-19): an Italian perspective. Journal of Clinical Pathology. 2020 Apr;

200. Villa E, Saccocci M, Messina A, Maffeo D, Pitì A, Bianchetti F, et al. [COVID-19 and coronary artery disease: selective and collaborative use of resources during public health crisis]. Giornale Italiano Di Cardiologia (2006). 2020 May;21(5):360–3.

201. Vitacca M, Nava S, Santus P, Harari S. Early consensus management for non-ICU ARF SARS-CoV-2 emergency in Italy: from ward to trenches. The European respiratory journal [Internet]. 2020; Available from: https://www.scopus.com/inward/record.uri?eid=2-s2.0-85083212250&doi=10.1183%2f13993003.00632-2020&partnerID=40&md5=513792112aa70b4851a1d3d5bd7ea76b

202. Volpato S, Landi F, Incalzi RA. A Frail Health Care System for an Old Population: Lesson form the COVID-19 Outbreak in Italy. The Journals of Gerontology Series A, Biological Sciences and Medical Sciences. 2020 Apr;

203. Zangrillo A, Beretta L, Silvani P, Colombo S, Scandroglio AM, Dell'Acqua A, et al. Fast reshaping of intensive care unit facilities in a large metropolitan hospital in Milan, Italy:

facing the COVID-19 pandemic emergency. Critical Care and Resuscitation: Journal of the Australasian Academy of Critical Care Medicine. 2020;

204. Zehender G, Lai A, Bergna A, Meroni L, Riva A, Balotta C, et al. Genomic characterization and phylogenetic analysis of SARS-COV-2 in Italy. Journal of Medical Virology [Internet]. 2020; Available from: https://www.scopus.com/inward/record.uri?eid=2-s2.0-85083104405&doi=10.1002%2fjmv.25794&partnerID=40&md5=b0919238f37745f0d70a997ea7960340

205. Zoia C, Bongetta D, Veiceschi P, Cenzato M, Di Meco F, Locatelli D, et al. Neurosurgery during the COVID-19 pandemic: update from Lombardy, northern Italy. Acta Neurochirurgica [Internet]. 2020; Available from: https://www.scopus.com/inward/record.uri?eid=2-s2.0-85082970377&doi=10.1007%2fs00701-020-04305-w&partnerID=40&md5=8b9d464f283df1012117b7edbc6870b6

Non-Italian articles included in the study

1. Bellizzi S, Fiamma M, Arru L, Farina G, Manca A. Covid-19: The daunting experience of health workers in Sardinia, Italy. Infection Control and Hospital Epidemiology. 2020 Apr;1–5.

2. Day M. Covid-19: identifying and isolating asymptomatic people helped eliminate virus in Italian village. BMJ (Clinical research ed). 2020;368:m1165.

3. Day M. Covid-19: Italy confirms 11 deaths as cases spread from north. BMJ (Clinical research ed). 2020;368:m757.

4. Day M. Covid-19: surge in cases in Italy and South Korea makes pandemic look more likely. BMJ (Clinical research ed). 2020;368:m751.

5. Dowd JB, Andriano L, Brazel DM, Rotondi V, Block P, Ding X, et al. Demographic science aids in understanding the spread and fatality rates of COVID-19. Proceedings of the National Academy of Sciences of the United States of America. 2020 Apr;

6. Etkind SN, Bone AE, Lovell N, Cripps RL, Harding R, Higginson IJ, et al. The role and response of palliative care and hospice services in epidemics and pandemics: a rapid review to inform practice during the COVID-19 pandemic. Journal of Pain and Symptom Management. 2020 Apr;

7. Giovanetti M, Angeletti S, Benvenuto D, Ciccozzi M. A doubt of multiple introduction of SARS-CoV-2 in Italy: A preliminary overview. Journal of Medical Virology [Internet]. 2020; Available from: https://onlinelibrary.wiley.com/doi/epdf/10.1002/jmv.25773

8. Giovanetti M, Benvenuto D, Angeletti S, Ciccozzi M. The first two cases of 2019-nCoV in Italy: Where they come from? Journal of Medical Virology. 2020;92(5):518–21.

9. Giwa AL, Desai A, Duca A. Novel 2019 coronavirus SARS-CoV-2 (COVID-19): An updated overview for emergency clinicians. Emergency medicine practice. 2020;22(5):1–28.

10. Goumenou M, Spandidos DA, Tsatsakis A. Possibility of transmission through dogs being a contributing factor to the extreme Covid-19 outbreak in North Italy. Molecular Medicine Reports. 2020;21(6):2293–5.

11. Khachfe HH, Chahrour M, Sammouri J, Salhab H, Makki BE, Fares M. An Epidemiological Study on COVID-19: A Rapidly Spreading Disease. Cureus. 2020 Mar;12(3):e7313.

12. Khafaie MA, Rahim F. Cross-Country Comparison of Case Fatality Rates of COVID-19/SARS-COV-2. Osong Public Health and Research Perspectives. 2020 Apr;11(2):74–80.

13. Khosrawipour V, Lau H, Khosrawipour T, Kocbach P, Ichii H, Bania J, et al. Failure in initial stage containment of global COVID-19 epicenters. Journal of Medical Virology. 2020 Apr;

14. Kinross P, Suetens C, Dias JG, Alexakis L, Wijermans A, Colzani E, et al. Rapidly increasing cumulative incidence of coronavirus disease (COVID-19) in the European Union/European Economic Area and the United Kingdom, 1 January to 15 March 2020. Eurosurveillance [Internet]. 2020;25(11). Available from: https://www.eurosurveillance.org/docserver/fulltext/eurosurveillance/25/11/eurosurv-25-11-1.pdf?expires=1591024650&id=id&accname=guest&checksum=7E08068347F7073E41D365605019ACAF

15. Kreutz R, Algharably EAE-H, Azizi M, Dobrowolski P, Guzik T, Januszewicz A, et al. Hypertension, the renin-angiotensin system, and the risk of lower respiratory tract infections and lung injury: implications for COVID-19. Cardiovascular Research. 2020;

16. Livingston E, Bucher K. Coronavirus Disease 2019 (COVID-19) in Italy. JAMA [Internet]. 2020; Available from: https://www.scopus.com/inward/record.uri?eid=2-s2.0-85082323068&doi=10.1001%2fjama.2020.4344&partnerID=40&md5=6abfd4cf8291604b69b591f74db423b6

17. Mallick R, Odejinmi F, Clark TJ. Covid 19 pandemic and gynaecological laparoscopic surgery: knowns and unknowns. Facts, Views & Vision in ObGyn. 2020 Apr;12(1):3–7.

18. Mavragani A. Tracking COVID-19 in Europe: Infodemiology Approach. JMIR public health and surveillance. 2020;6(2):e18941.

19. Paterlini M. Covid-19: Over 300 Italian doctors and scientists call for more testing. BMJ (Clinical research ed). 2020;368:m1274.

20. Paterlini M. On the front lines of coronavirus: The Italian response to covid-19. The BMJ [Internet]. 2020;368. Available from: https://www.scopus.com/inward/record.uri?eid=2-s2.0-85081991318&doi=10.1136%2fbmj.m1065&partnerID=40&md5=4e4868061d0b8b0e5429ab8ff13a5dee

21. Paterlini M. Lockdown in Italy: personal stories of doing science during the COVID-19 quarantine. Nature. 2020 Apr;

22. Rosenbaum L. Facing Covid-19 in Italy - Ethics, Logistics, and Therapeutics on the Epidemic's Front Line. The New England journal of medicine [Internet]. 2020; Available from: https://www.scopus.com/inward/record.uri?eid=2-s2.0-85083017811&doi=10.1056%2fNEJMp2005492&partnerID=40&md5=21deddf22aaa137a7edaeb62220a03c5

23. Shojaee S, Pourhoseingholi MA, Ashtari S, Vahedian-Azimi A, Asadzadeh-Aghdaei H, Zali MR. Predicting the mortality due to Covid-19 by the next month for Italy, Iran and

South Korea; A simulation study. Gastroenterology and Hepatology from Bed to Bench. 2020;13(2):177–9.

24. Sjödin H, Wilder-Smith A, Osman S, Farooq Z, Rocklöv J. Only strict quarantine measures can curb the coronavirus disease (COVID-19) outbreak in Italy, 2020. Eurosurveillance [Internet]. 2020;25(13). Available from: https://www.scopus.com/inward/record.uri?eid=2-s2.0-85083056872&doi=10.2807%2f1560-7917.ES.2020.25.13.2000280&partnerID=40&md5=61f9051c11b3f961eea6a13e0947b676

25. Solnica A, Barski L, Jotkowitz A. Allocation of scarce resources during the COVID-19 pandemic: A Jewish ethical perspective. Journal of Medical Ethics [Internet]. 2020; Available from: https://www.scopus.com/inward/record.uri?eid=2-s2.0-85083274267&doi=10.1136%2fmedethics-2020-106242&partnerID=40&md5=0a228c40dbfe0a0cde6006e2ebfc2219

26. Tobías A. Evaluation of the lockdowns for the SARS-CoV-2 epidemic in Italy and Spain after one month follow up. Science of the Total Environment [Internet]. 2020;725. Available from: https://www.scopus.com/inward/record.uri?eid=2-s2.0-85083111626&doi=10.1016%2fj.scitotenv.2020.138539&partnerID=40&md5=d766056d5f7de51674e83d86e2c38a19

27. Tuite AR, Ng V, Rees E, Fisman D. Estimation of COVID-19 outbreak size in Italy. The Lancet Infectious Diseases [Internet]. 2020; Available from: https://www.scopus.com/inward/record.uri?eid=2-s2.0-85082193464&doi=10.1016%2fS1473-3099%2820%2930227-9&partnerID=40&md5=09180cdd4e6e9b18e9b55a740c3daf81

28. Verelst F, Kuylen E, Beutels P. Indications for healthcare surge capacity in European countries facing an exponential increase in coronavirus disease (COVID-19) cases, March 2020. Eurosurveillance [Internet]. 2020;25(13). Available from: https://www.scopus.com/inward/record.uri?eid=2-s2.0-85083045313&doi=10.2807%2f1560-7917.ES.2020.25.13.2000323&partnerID=40&md5=ad15a39b68fd1fa951335dea5e87bb63

29. Volpert V, Banerjee M, Petrovskii S. On a quarantine model of coronavirus infection and data analysis. Mathematical Modelling of Natural Phenomena [Internet]. 2020;15. Available from: https://www.scopus.com/inward/record.uri?eid=2-s2.0-85082884865&doi=10.1051%2fmmnp%2f2020006&partnerID=40&md5=5653bc3d6d6dd7c822805eeb4756e78f

30. Walker A, Hopkins C, Surda P. The use of google trends to investigate the loss of smell related searches during COVID-19 outbreak. International Forum of Allergy & Rhinology. 2020 Apr;

31. Yuan J, Li M, Lv G, Lu ZK. Monitoring Transmissibility and Mortality of COVID-19 in Europe. International journal of infectious diseases: IJID: official publication of the International Society for Infectious Diseases. 2020 Mar;

32. Zhang X, Ma R, Wang L. Predicting turning point, duration and attack rate of COVID-19 outbreaks in major Western countries. Chaos, Solitons, and Fractals. 2020 Apr;109829.

33. Zheng MH, Boni L, Fingerhut A. Minimally Invasive Surgery and the Novel Coronavirus Outbreak: Lessons Learned in China and Italy. Annals of surgery [Internet]. 2020; Available from: https://www.scopus.com/inward/record.uri?eid=2-s2.0-85082468884&doi=10.1097%2fSLA.0000000000003924&partnerID=40&md5=6e2ba64c7f7198bb1e5c0c5e1cb186d9
